# Chemical Diversity and Biological Activity of Secondary Metabolites from Soft Coral Genus *Sinularia* since 2013

**DOI:** 10.3390/md19060335

**Published:** 2021-06-11

**Authors:** Xia Yan, Jing Liu, Xue Leng, Han Ouyang

**Affiliations:** 1 Li Dak Sum Yip Yio Chin Kenneth Li Marine Biopharmaceutical Research Center, Department of Marine Pharmacy, College of Food and Pharmaceutical Sciences, Ningbo University, Ningbo 315800, China; yanxia@nbu.edu.cn (X.Y.); liujingsally@163.com (J.L.); lxue1520@163.com (X.L.); 2 Institute of Drug Discovery Technology, Ningbo University, Ningbo 315211, China

**Keywords:** soft coral, *Sinularia*, secondary metabolites, bioactivity

## Abstract

*Sinularia* is one of the conspicuous soft coral species widely distributed in the world’s oceans at a depth of about 12 m. Secondary metabolites from the genus *Sinularia* show great chemical diversity. More than 700 secondary metabolites have been reported to date, including terpenoids, norterpenoids, steroids/steroidal glycosides, and other types. They showed a broad range of potent biological activities. There were detailed reviews on the terpenoids from *Sinularia* in 2013, and now, it still plays a vital role in the innovation of lead compounds for drug development. The structures, names, and pharmacological activities of compounds isolated from the genus *Sinularia* from 2013 to March 2021 are summarized in this review.

## 1. Introduction

**Figure 1:**
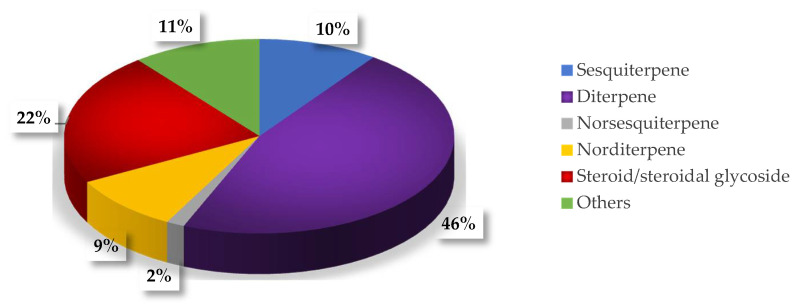
Compounds from genus *Sinularia* with different ratios of structural types.

Secondary metabolites from marine organisms represented a plentiful source of structurally diverse and natural bioactive products. The soft corals of genus *Sinularia* (phylum Cnidaria, class Anthozoa, subclass Octocorallia, order Alcyonacea, family Alcyoniidae) inhabiting the coral reefs or rocks in shallow water constitutes a dominant portion of the biomass in the tropical coral reef systems in the world. There are more than 90 species of *Sinularia*, and more than 50 species have been chemically evaluated from 1975 to 2013 [1]. As reported, *Sinularia* is well-known to produce a wealth of diverse and complex secondary metabolites, such as sesquiterpenes (10%), diterpenes (46%), norsesquiterpenes (2%), norditerpenes (9%), steroids/steroidal glycosides (22%), and other types (11%) (Figure 1) [1,2,3]. These metabolites exhibit a wide range of biological activities, such as cytotoxic, anti-inflammatory, antifouling, and anti-microbials [4,5,6,7,8,9,10,11,12,13,14].

There are some published literature reviews on the genus *Sinularia*. For example, Liang et al. [15] reviewed the secondary metabolites obtained from *Sinularia* and summarized the different types of chemical structures and/or biological investigations within a literature survey from 1975 to April 2013. Chen et al. [1] reviewed the terpenes from the soft coral of the genus *Sinularia*, highlighting their novel chemistry and pharmacological activities. Sheu et al. [16] reviewed the structures, names, and bioactivities of 75 marine diterpenoids from octocorals possessing a hydroperoxy group, of which 41 compounds exhibited potential biomedical activities. Lakshmi et al. [17] summarized the chemical constituents of ≈40 *S.* sp. covering a period of 1975–2004. Rodrigues et al. [18] reviewed the new isolated cembranoid derivatives from species of genera *Sarcophyton*, *Sinularia*, and *Lobophytum* as well as their biological properties during 2016–2018. Especially, the current review focuses on the chemical structures and the biological activities of secondary metabolites obtained from soft coral genus *S.* since 2013; the Scifinder and Web of Science databases were used for research (Table 1).

## 2. Chemistry and Bioactivity of Secondary Metabolites from Genus *Sinularia*

**Scheme 1:**
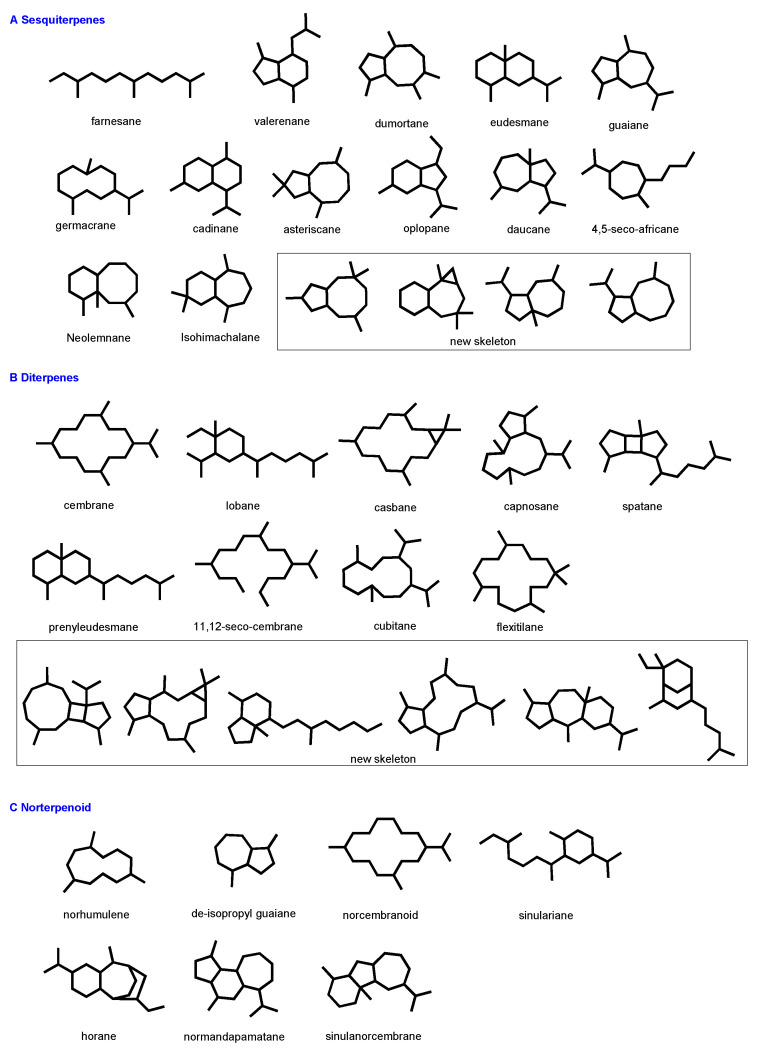
Various carbon skeletons of terpenoids from soft coral of genus *Sinularia* since 2013.

Genus *Sinularia* is one of the most widely distributed soft coral genera over the oceans. Many of them were chemically investigated. Especially *Sinularia* collected from the South China Sea, Taiwan, Vietnam, and Malaysia have provided diverse structures in recent years [4,36,38,59]. On the investigations of the chemical constituents from marine soft coral genus *Sinularia*, about 100 research papers have been published, and about 249 new compounds have been isolated and identified from more than 30 species in recent years, such as *S. brassica*, *S. depressa*, *S. erecta*, *S. flexibilis*, *S. humilis*, *S. kavarattiensis*, *S. leptoclados*, *S. nanolobata*, *S. polydactyla*, *S. scabra,* and other unidentified *S.* sp. (Table 1). Terpenoids and steroids/steroidal glycosides are the prominent metabolites in *S.* sp. Terpenoids from the genus *Sinularia* could be classified into sesquiterpenoids, diterpenes, norsesquiterpenes, norditerpenes, etc. (Scheme 1). Steroids/steroidal glycosides are mainly composed of normal steroids and seco-steroids. Marine-derived terpenoids have attracted the interest of natural product chemists around the world, leading to the discovery of structurally diverse metabolites, featuring many novel scaffolds equipped with a diverse array of functionality. Especially, some microbial origin terpenoids from sponges and tunicates were characterized by rare and unprecedented structures and biological activities [89,90].

### 2.1. Terpenoids

#### 2.1.1. Sesquiterpenes and Bioactivities

**Figure 2:**
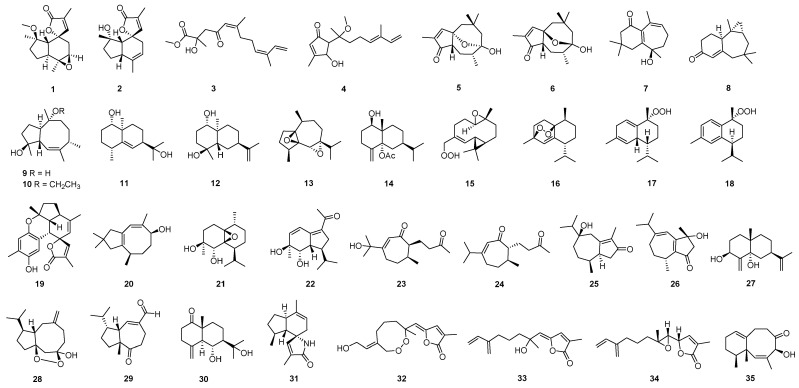
Sesquiterpenes isolated from the genus *Sinularia*.

A farnesyl diphosphate (FPP, C15) is regarded as the same biosynthetic precursor of sesquiterpene. Sesquiterpenes from *S.* sp. include chain and ring structures. The ring structures are formed from one or more cyclizations of FPP by FPP cyclase. Various carbocation intermediates formed after cyclization may need further cyclization, hydrogen anion migration, methyl transfer, or Wagner–Meerwein rearrangement to remove protons or absorb protons to form the final products. In recent years, about 35 new sesquiterpenes (Figure 2) have been isolated from the genus *Sinularia*. According to the carbon skeletons, it can be classified into 17 types, including four new carbon skeletons. (Figure 1). Various activities about these new sesquiterpenes, such as cytotoxicity [78], anti-inflammatory [76], antimicrobial [51], and antimalarial [10], were reported (Table 2).

Four new sesquiterpenes, sinularianins C–F (**1**–**4**), were separated from a South China Sea soft coral *S.* sp. [78]. **1** and **2** were valerenane-type sesquiterpenes, and **3** and **4** were farnesane-type sesquiterpenes. Although compounds **3** and **4** displayed a quite different skeleton from that of **1** and **2**, they are related to each other. From a biosynthetic aspect, sinularinins C–D (**1**–**2**), and F (**4**) could be generated from sinularinin E (**3**) via different reaction cascades [78], including dehydration, Diels Alder cyclization, Michael addition, epoxidation and dehydration, and aldol condensation.

Chemical examination of the soft coral *S. capillosa* from the Sanya Bay resulted in the isolation of eight new sesquiterpenoids named capillosananes S–Z (**5**–**12**) [76]. The CD effects and Mosher method were determined for the assignment of their absolute configurations. Capillosananes S–U (**5**–**7**) presented as the novel carbon skeletons with bicyclo[3.6.0] and bicyclo[4.5.0] systems, while capillosanane V (**8**) was characteristic of an unprecedented tricyclic skeleton [76]. Capillosananes W–X (**9**, **10**) were assigned to the unusual dumortane-type sesquiterpenes, and capillosananes Y–Z (**11**, **12**) were eudesmane-type sesquiterpenes. It was the first report of dumortane analogues from marine organisms [76].

A new guaiane sesquiterpene (**13**) was isolated from the soft coral *S.* *kavarattiensis*, which was collected from the Mandapam coast of the Indian Ocean [70]. The relative stereochemistry of the methyl group and epoxy groups in compound **13** was confirmed in search of the most stable conformer of sesquiterpenoid by molecular mechanics (UFF) and density functional theory calculations, in addition to NOESY correlations [70].

A new eudesmane sesquiterpenoid 5α-acetoxy-4(14)eudesmene-1β-ol (**14**) was isolated from the octocoral *S. gaweli* [64].

Three cadinane-type sesquiterpenoids, endoperoxide (**16**) and hydroperoxides (**17**, **18**), were obtained from an Okinawan soft coral of the *Sinularia* species [51]. The biogenetic pathways of **16**, **17** were proposed. By a first cyclization, a 1,3-hydride shift, a second cyclization, and then deprotonation, δ-Cadinene was formed from farnesyl pyrophosphate [51]. δ-Cadinene gave cadinane-type endoperoxy and hydroperoxy sesquiterpenoids **16**, **17** through a 1,3-hydride shift, and/or deprotonation and a reaction with singlet oxygen (Diels–Alder reaction or ene reaction) [51].

An unprecedented highly fused benzosesquiterpenoid verrubenzospirolactone (**19**) and a new asteriscane-type sesquiterpenoid 10-deoxocapillosanane D (**20**) were isolated from a South China Sea collection of the soft coral *S. verruca* [49]. Compound **19** was a novel skeleton that could be derived from capillobenzopyranol via a series of oxidation and cyclization reactions [49].

A new cadinane-type sesquiterpene and a new oplopane-type sesquiterpene, nanolobatols A and B (**21** and **22**), were isolated from the Vietnamese soft coral *S. nanolobata* [44].

Two 4,5-seco-africane-type sesquiterpenes, molestin A (**23**) and *epi*-gibberodione (**24**), and two guaiane-type sesquiterpenes, molestins B–C (**25**, **26**), were isolated from the Paracel Islands soft coral *S.* cf. *molesta* [40]. Compound **26** displayed strong inhibitory activities against PTP1B [40].

A new eudesmane sesquiterpenoid 3*β*,5*α*-dihydroxyeudesma-4(15),11-diene (**27**) was isolated from the metabolites of a Vietnamese soft coral *S. erecta* [38]. It exhibited selective cytotoxicity against the A549 cell line [38].

Three new sesquiterpenoids, sinuketal (**28**) and sinulins A and B (**29** and **30**), were isolated from the Xisha soft coral *S.* sp [10]. Compound **28** was the first example of marine-originated isopropyl branche undecane sesquiterpenoid, which possessed an unprecedented a bicyclo[6.3.0] undecane carbon skeleton with unique endoperoxide moiety, and compound **30** was a eudesmane sesquiterpenoid [10]. The relative configuration of **28** was established by a nuclear Overhauser effect spectroscopy (NOESY) experiment in combination with conformational analysis and computational approaches such as the density functional theory-NMR (DFT-NMR) method. The endoperoxide group should be on the same side of the cyclooctane ring, owing to unfavorable distortions on the basis of molecular model analysis [10].

A novel aza-spirocyclic valerenane sesquiterpenoid sinulaspirolactam A (**31**) was isolated from the soft coral *S.* sp. [31]. It was the first example of valerenane sesquiterpenoid bearing an aza-spiro[4.5] ring moiety [31]. The biogenetic pathway of **31** could be plausibly proposed. It was originated from the successive condensation of IPP and DMAPP. Valerendiene synthase (VoVDS) and then catalyzed by the cyclization of FPP into an intermediate, which underwent a series of subsequent conversions, including oxidation, double bond migration, addition, amination, and dehydration to generate **31** [31]. The cytotoxic activities were evaluated against SW480, MCF-7, HepG2, HeLa, and PANC-1 cell lines. However, **31** showed no obvious activity [31].

Three farnesane sesquiterpenoids sinulatumolin A–C (**32**–**34**) and a new neolemnane sinulatumolin E (**35**) were isolated from the South China Sea soft coral *S. tumulosa* [8]. Sinulatumolin A (**32**) represented the first example of sesquiterpene bearing an eight-membered cyclic peroxide ring from soft coral, and **34** represented the second furanosesquiterpenoid with a 2-methylfuran-3(2H)-one moiety [8]. Compounds **32**, **34**, and **35** displayed significant TNF-α inhibitory activity [8].

The bioactivities of new sesquiterpenes discovered from *S.* sp. were summarized in Table 2. It is revealed that sesquiterpene showed no significant cytotoxicity, but some of them have promising anti-inflammatory activity. These sesquiterpenes with the hydroperoxyl group showed attractive structures and a diversity of biological activities, such as anti-inflammatory activities (compounds **16**–**18** and **32**) and antimalarial activities compound **28**). As for compound **15**, no activity screening was performed.

#### 2.1.2. Diterpenes

**Figure 3:**
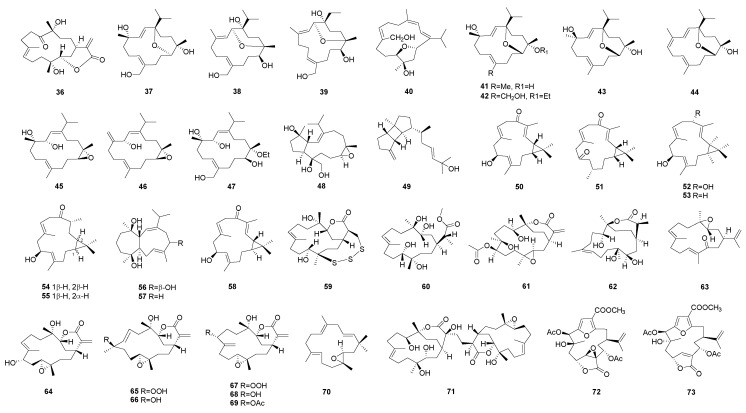

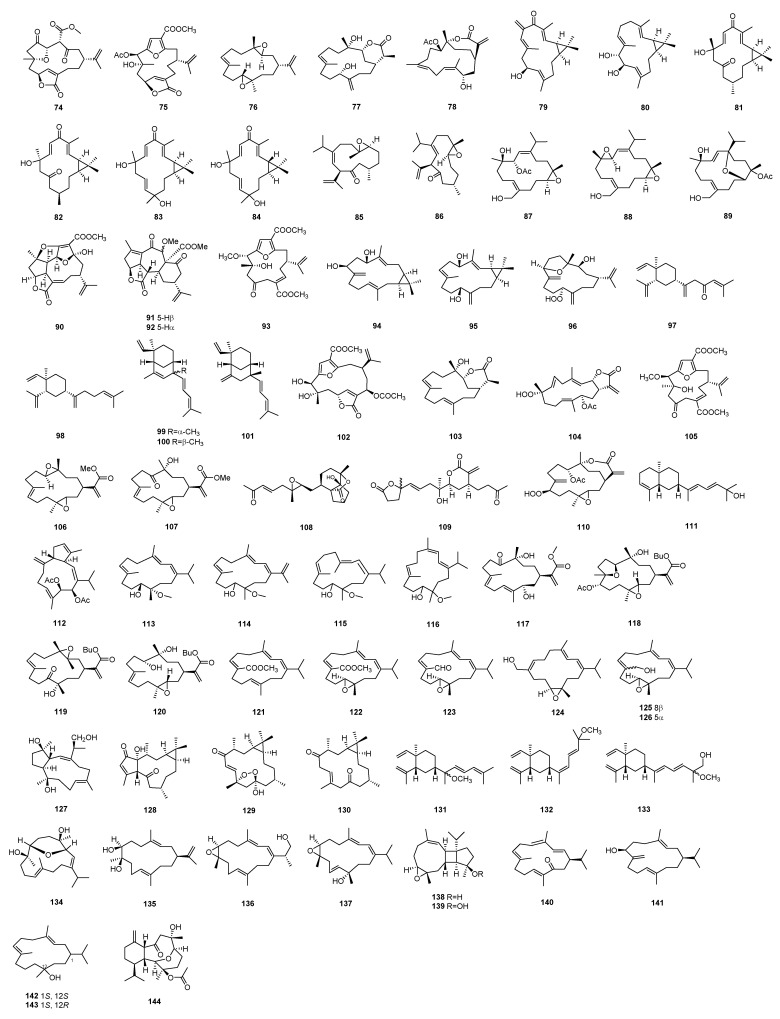
Diterpenoids isolated from the genus *Sinularia*.

Diterpenes are synthesized by four isoprene units, and their basic structure starts with geranylgeranyl-PP (GGPP, C20). GGPP turns into a highly active carbocation intermediate catalyzed by diterpene synthase, which induced a series of cyclization reactions to form diterpenoid skeletons. The diterpenoid intermediate modified by secondary enzymatic reactions, such as hydroxylation, peroxidation, methylation, acylation, or lytic rearrangement, to produce a final product with more structural diversity and biological activity. About 109 diterpenes with different structures belonging to different chemical classes were reported from the *Sinularia* species in the past 8 years (Figure 3). In accordance with sesquiterpenes in this genus, the structures of diterpenoids are also variable, which could be categorized as many types [1], such as cembrane-type, casbane-type, lobane-type, and other types, including some new carbon skeletons. Among them, the cembrane-type diterpenoid has the most diverse structural variations with a multitude of functional groups (lactone, epoxide, furan, ester, aldehyde, hydroxyl, carboxyl moieties) and cyclizations [18], which could be classified into many subtypes, such as isopropyl cembranes, isopropenyl cembranes, cembranolides (5/6/7-membered lactone), furanocembranoids, and biscembranoids. Diterpenoids have been reported to display a variety of biological activities, including anti-inflammatory activity [85], cytotoxicity [72], antifouling activity against bryozoan and barnacle [14], antimicrobial activity [53], PTP1B inhibitory activity [34], anti-Aβ aggregation activity [32], α-glucosidase inhibitory activity [88], and so on.

A new cembranoidal diterpene flexibilin D (**36**) with a 6-membered lactone ring was isolated from the Taiwan soft coral *S. flexibilis* [85]. It was found to significantly inhibit the accumulation of the pro-inflammatory iNOS and COX-2 proteins of the lipopolysaccharide (LPS)-stimulated RAW264.7 macrophage cells [85].

Twelve new cembranoids sinulariols T−Z_4_ (**37**–**47**) and a new capnosane diterpene sinulariol Z_5_ (**48**) were separated from *S. rigida* [14], which was collected in Sanya Bay, Hainan Island of China. Compound **43** displayed dose-dependency and showed significant inhibition against the larval settlement of the *barnacle Ba. amphitrite* and moderate inhibition against *Bu. neritina* [14]. In addition, compound **43** showed a high therapeutic ratio for the inhibition of *Ba. amphitrite* [14].

Spatane diterpenoids are characterized by a unique tricyclic [5.3.0.0^26^] decane ring [91]. A new spatane diterpenoid, leptoclalin A (**49**), was isolated from a cultured soft coral *S. leptoclados* [79]. A computer-modeled 3D structure of **49** was generated, and MM2 force-field calculations produced the energy minimization, which was compatible with the NOE correlations. Compound **49** was found to show weak cytotoxicity toward the growth of T-47D and K-562 (human chronic myelogenous leukemia) tumor cells [79].

The casbanes are a small group of diterpenoids strictly related to the cembrane skeleton and characterized by the presence of a dimethyl-cyclopropyl moiety fused to the 14-membered ring [77]. Six new casbane diterpenoids, sinularcasbanes A–F (**50**–**55**) were isolated from a South China Sea *S.* sp. soft coral [77]. These compounds were tested for cytotoxicity against a panel of tumor cell lines, but they showed no cytotoxic activity [77]. Compounds **51** and **54** showed moderate inhibition against lipopolysaccharide (LPS)-induced nitric oxide (NO) production [77].

Two novel diterpenoids, sinularbols A (**56**) and B (**57**) with a novel sinularborane-type carbon skeleton (3,9-cyclized cembranoid), were isolated from the Taiwan soft coral *S. arborea* [75]. The relative configuration of **56** was elucidated from the NOESY experiment and was found to be compatible with the computer modeling results. Compound **57** displayed a moderate inhibitory effect on the generation of superoxide anion [75].

One novel nine-membered macrocyclic polysulfur cembranoid lactone, sinulariaoid A (**59**); and three new multioxygenated cembranoids, sinulariaoid B (**60**), sinulariaoid C (**61**), and sinulariaoid D (**62**), were isolated from the soft coral *S.* sp. [72], which was collected off of Sanya Bay in the South China Sea. The absolute stereochemical structure of sinulariaoid A (**59**) was elucidated by single crystal X-ray diffraction analysis, and it was the first reported nine-membered macrocyclic polysulfur cembranoid from soft coral [72]. The cembranoid carbon skeleton is a logical biosynthetic precursor, and it yields many types of cembrane diterpenoid lactones via lactonization, hydrogenation, ring-opening reaction, methyl esterification, oxidation, acid-catalyzed hydrolysis, and so on [72]. The GSH was speculated to be the sulfur donor, and a CYP450 monooxygenase and glutathione S-transferase (GST) play a key role in gliotoxin C–S bond formation [72]. **59** was shown to be cytotoxic toward some cancer cell lines.

A new cembrane-type diterpenoid, arbolide C (**63**), was obtained from the Taiwan soft coral [69], and it was found to display in vitro anti-inflammatory activities with an inhibitory effect on the release of elastase by human neutrophils [69].

Eight new terpenoids, including six α-methylene-δ-lactone-bearing cembranoids (**64**–**69**), a flexitilane-type diterpenoid (**70**), and a biscembranoid (**71**), were isolated from the South China Sea soft coral *S. flexibilis* [65]. The structure of **71** was confirmed by X-ray diffraction analyses. Epoxyfexibilene (**70**) represents the second 15-membered macrocyclic diterpenoid being discovered from marine sources, and sinulafexiolide L (**71**) is the third member of the rare cembrane dimers connected through a C–C single bond [65].

Two new rare C-7, C-8 vicinal diol-based cembrane diterpenoids, (−)-leptodiol acetate (**72**) and sinulacembranolide A (**73**), were isolated from the Taiwan soft coral *S. gaweli* [64]. Compound **73** was found to significantly reduce the levels of iNOS with no cytotoxicity to RAW264.7 macrophage cells [64].

New cembranoids 4-carbomethoxyl-10-epigyrosanoldie E (**74**) 7-acetylsinumaximol B (**75**) diepoxycembrene B (**76**), dihydromanaarenolide I (**77**), and isosinulaflexiolide K (**78**) were isolated from cultured soft corals *S. sandensis* and *S. flexibilis* [63]. The absolute configurations of **74** and **78** were further confirmed by single-crystal X-ray diffraction analysis. It should be noticed that the hypothesis (that cembrane diterpenes possessing an absolute configuration of an isopropyl group at C1 obtained from Alcyonacean soft corals belong to the α series, whereas analogues isolated from Gorgonacean corals belong to the β series) is not applicable for a small number of cembranoids. Compound **78** was found to significantly reduce the levels of iNOS and COX-2 [63]. It was proposed that the presence of one seven-membered lactone functional group was critical for anti-inflammatory activity [63].

Six new casbane diterpenoids, sinularcasbanes G–L **(79**–**84**), were separated from the soft coral *S.* sp. [62], which was collected from Dongluo Island, Hainan Province of China. However, these compounds showed no cytotoxicity against ten human cancer cell lines (H1975, U937, K562, BGC823, MOLT-4, MCF-7, A549, HeLa, HL60, and Huh-7).

Two new cubitane-type diterpenoids, nanoculones A and B (**85** and **86**) and three new cembranoids, nanolobols A–C (**87**–**89**), were isolated from Taiwan soft coral *S. nanolobata* [59]. Compound **86** could effectively reduce the levels of LPS-stimulated NO production in activated RAW264.7 cells. These compounds were not cytotoxic against P388, K562, and HT-29 cell lines [59]. Considering their prior work on the same species collected in different locations of Taiwan water, it is noteworthy that the metabolites from soft corals, even the skeletons, might vary with their geographical location [59].

Four new diterpenoids, pambanolides A–C (**90**–**93**), were isolated from the soft coral *S. inelegans* [58], which was collected in the Gulf of Mannar on the southern coast of India. A biosynthetic pathway has been proposed for the formation of pambanolides A (**90**). It could be obtained from the precursor metabolite mandapamate, via oxa-Michael addition and intramolecular rearrangement [58]. Compounds **91** and **92** showed weak activity against the DU145 and A549 cancer cell lines [58].

A solvent extract of *S. polydactyla* resulted in the isolation of two new casbane diterpenes: sinularcasbane M (**94**) and sinularcasbane N (**95**). Compounds were elucidated based on spectroscopic analyses; the absolute configuration was confirmed by X-ray analysis [57].

A cembranoid sinulerectol C (**96**) with a hydroperoxy group was separated from soft coral *S. erecta* [3], which was collected off the coast of Dongsha Atoll. Compound **96** showed cytotoxicity toward the K-562 cell line. Moreover, it exhibited potent inhibitory activity against elastase release [3].

Two new lobane diterpenoids, prenyl-α-elemenone (**97**) and *ent*-prenyl-β-elemene (**98**), were obtained from Bornean soft coral *S.* sp. [53]. Cytotoxicity assays against B16-F10 and HT-29 cells displayed no activity. However, compound **97** exhibited inhibition against *S. aureus* [53].

Three new diterpenes with a new carbon skeleton, xishacorenes A−C (**99**–**101**), featuring an undescribed bicyclo[3.3.1]nonane nucleus bearing 1-vinyl and 13-[(*E*)-4-methylpenta-1,3-dien-1-yl] alkyl chains were isolated from the Xisha soft coral *S. polydactyla* [41]. The new skeleton was structurally related with lobane-type diterpenes by sharing some common moieties, such as the cyclohexane ring bearing a vinyl group and a conjugated diene side chain [41]. The research team proposed that an electron delivery from the Δ^10/12^ olefinic head to the activated hydroxyl tail accomplished the cyclization of a six-member ring [41]. These three compounds were found to promote the ConA-induced T lymphocytes proliferation with dose-dependency and had no activity on LPS induced B lymphocytes proliferation.

A new furanocembranolide molestin E (**102**) was isolated from the Paracel Islands soft coral *S.* cf. *molesta* [40], and it exhibited cytotoxicities against HeLa and HCT-116 cell lines [40].

A new cembrane *ent*-sinuflexibilin D (**103**) was isolated from a Bornean soft coral *S. flexibilis* [37]. Compound **103** showed cytotoxicity against the S1T cell line [37].

A new cembranoid sinularolide F (**104**) was isolated from the Bornean soft coral *S.* sp. [36]. It showed potential anti-inflammatory activities against lipopolysaccharide-stimulated RAW 264.7 through inhibiting NO synthesis by reducing the expression of iNOS protein. In addition, compound **104** exhibited apoptosis activity against HL-60 cells. The expressions of apoptotic proteins suggested that **104** triggered the up-regulation of Bax, the downregulation of Bcl-xL, and the activation of caspase-3 in the apoptosis mechanism [36].

Five new cembranoid-related diterpenoids, flexibilisins D and E (**106** and **107**), secoflexibilisolides A and B (**108** and **109**), and flexibilisolide H (**110**) were obtained from Taiwan soft coral *S. flexibilis* [35]. Compound **108** possessed an unusual skeleton that could be biogenetically derived from cembranoid flexibilisolide D, which is a known compound that was also isolated from this coral. After oxidative cleavage, Michael addition, condensation, rearrangement, and reduction, flexibilisolide D could be converted to secoflexibilisolide A. Compound **109** could be derived from sinuflexolide via oxidative cleavage of the diol groups, epimerization of the vinyl alcohol, and subsequent esterification. These compounds were nontoxic toward cancer cell lines P-388, K-562, and HT-29 [35].

A new prenyleudesmane type diterpene sinupol (**111**) and a new capnosane type diterpenoid sinulacetate (**112**) were isolated from the Xisha soft coral *S. polydactyla* [34]. The time-dependent density functional theory electronic circular dichroism (TDDFT ECD) calculation was applied to establish their absolute configurations [34]. Compound **111** was proposed to be a cyclization product of fuscol, and a capnosane diterpenoid, sinulacetate (**112**), with 5:11-fused carbobicyclic skeleton, could be derived from the cembranoid [34]. These two new compounds exhibited promising inhibitory activity against protein tyrosine phosphatase 1B (PTP1B) [34].

Four new cembranoids (**113**–**116**) were isolated from the South China Sea soft coral *S.* sp. [32]. The absolute configuration of **113** was established by X-ray diffraction analysis. Compounds **113** and **115** displayed moderate inhibitory activity against Aβ_42_ aggregation [32]. The binding mode of **113** with Aβ_42_ monomer was predicted by molecular docking. In addition, compounds **113**–**115** did not show cytotoxicity against human tumor cell lines (SH-SY5Y, MDA-MB-426, A549, Hep3B, and HT-29) [32].

A new cembrane-type diterpenoid, sinulaflexiolide P (**117**), was isolated from a Bornean soft coral *S. flexibilis* [29]. It was screened against six fungal strains: *Fusarium moniliforme* (NJM 8995), *F. oxysporum* (NJM 0179), *F. solani* (NJM 8996), *Haliphthoros milfordensis* (IPMB 1603), *H. sabahensis* (IPMB 1402), and *Lagenidium thermophilum* (IPMB 1401). It exhibited promising inhibitory activity against *H. sabahensis*, *L. thermophilum*, and *H. milfordensis* [29].

Three novel cembranoid esters xidaosinularide A–C (**118**–**120**) featuring an n-butyl alcohol moiety were obtained from Hainan soft coral *S. flexibilis* [27]. Compound **118** reduced the levels of TNF-α in lipopolysaccharide (LPS)-stimulated RAW 264.7 cells [27].

Six new cembrane type diterpenoids xiguscabrates A and B (**121** and **122**), xiguscabral A (**123**), xiguscabrols A and B (**124** and **125**), and 8-*epi*-xiguscabrol B (**126**) were isolated from the South China Sea soft coral *S. scabra* [26]. Compounds **122** and **124**–**126** exhibited strong inhibitory activity on the proliferation of Con A-induced T lymphocyte cells [26]. It was suggested that the position and the number of hydroxyls might influence the toxicity of cembranoids, while it could not affect the immunological activity [26].

A new diterpenoid with an unusual capnosane skeleton, sinuhumilol A (**127**), was isolated from the South China Sea soft coral *S. humilis* [23]. However, it showed no obvious cytotoxicity against a series of tumor cell lines, including A549 (lung adenocarcinoma), HT-29 (colonic carcinoma), SNU-398 (hepatocellular carcinoma), and Capan-1 (pancreatic carcinoma) [23].

A novel diterpenoid, sinueretone A (**128**), featuring an unprecedented tricyclo [12.1.0.0^5,9^] pentadecane carbon framework, and two new casbane-type diterpenoids sinuereperoxide A (**129**) and 10-oxo-3,4,11,12-tetrahydrodepressin (**130**) were isolated from the South China Sea soft coral *S. erecta* [7]. Analysis of the X-ray data unambiguously confirmed the planar structure of **129** and the determination of its absolute configuration. Compounds **128** and **129** displayed anti-inflammatory activity of lipopolysaccharide (LPS)-induced tumor necrosis factor (TNF)-α protein release in RAW264.7 macrophages [7]. Compound **129** showed anti-inflammatory activity, indicating that the peroxide bridge might be helpful for the activity. Both of them exhibited no obvious cytotoxicity against RAW264.7 cells [7].

Three new lobane-type diterpenes, 13-methoxyloba-8,10,15(16),17(18)-tetraene (**131**), 8,10,13(15)*Z*,16*E*-lobatetraene (**132**), and 19-hydroxy-lobatetraene (**133**) were isolated from the South China Sea soft coral, but they are inactive on antibacterial, PTP1B inhibitory, and immunological activities [21].

Four new cembranoids, humilisins A−D (**134**−**137**), and two new uncommon diterpenoids humilisins E and F (**138** and **139**) with new carbon skeletons were isolated from the South China Sea soft coral *S. humilis* [4]. Humilisin A (**134**) was the first cembranoid with an ether linkage between C-3 and C-7. Humilisins E and F (**138** and **139**) possessed a tetradecahydrocyclopenta[3′,4′]cyclobuta[1′,2′:4,5]cyclonona [1,2-*b*]-oxirene ring system [4]. Compound **139** displayed a significant inhibitory effect on LPS-induced inflammatory response (NO production) in BV-2 microglial cells, and the level of NO decreased [4].

Four new cembranoids, sinulacrassins A−C (**140**–**142**) and *ent*-xishaflavalin G (**143**), were yielded from the South China Sea soft coral *S. crassa* [88]. The bioassay results revealed that compound **141** was a novel α-glucosidase inhibitor and nontoxic toward human normal hepatocyte (LO2) cells [88].

A eunicellin-based diterpenoid multifloralin (**144**) was isolated from the extract of the South China Sea soft coral *S. multiflora* [28]. It showed potent antifouling activity against barnacle *Balanus albicostatus* [28].

As shown in Table 3, diterpenoids from *S.* sp. were found to be of promising bio-functional diversities. The biological properties reported in several works were attributed to the variability of chemical structures. It could be concluded that nearly all the bioactive diterpenoids possess oxygenic rings or hydroxy groups, and the position of the oxygenic ring, as well as the position and number of hydroxyls, might influence the activities. For example, the presence of one seven-membered lactone functional group at position C1 is critical for the anti-inflammatory action of this class of compounds [63]. In particular, the multisulfide moiety of sinulariaoid A (**59**) played an important role in the cytotoxic bioactivities. The diterpenoids with a hydroperoxyl group also showed significant anti-inflammatory activity or cytotoxicity.

#### 2.1.3. Norterpenoid

**Figure 4:**
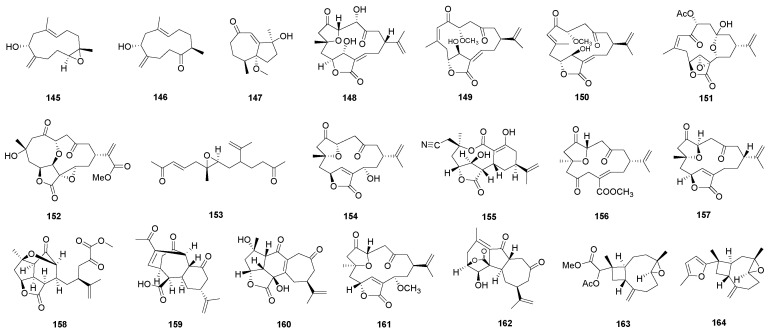
Norterpenoids isolated from genus *Sinularia*.

The mechanisms leading to the occurrence of norterpenoids are not well understood, but they may include the production of anionic and radical intermediates along with competitive transannular carbon-to-carbon bond-forming reactions [18]. Norterpenoids could be commonly considered as one of the chemotaxonomic markers of soft corals of genus *Sinularia*. To date, several novel norsesquiterpenoids and norditerpenoids have been isolated and structurally elucidated from *Sinularia* species. The structures of norsesquiterpenoids could be variable as sesquiterpenoids, but there were not many reported. Up until now, about 10 norsesquiterpenoids were obtained from *S.* sp.; to the best of our knowledge, three of them were found since 2013 (Figure 4). Two were uncommon norhumulene-type norsesquiterpenoids sinuhirtins A (**145**) and B (**146**), which were isolated from the South China Sea soft coral *S. hirta* [30]. The norhumulene was suggested to be the precursor. Molestins D (**147**) was the first example of a norsesquiterpene with a de-isopropyl guaiane skeleton isolated from the genus *Sinularia* [40], and it showed significant inhibitory activities against protein tyrosine phosphatase 1B (PTP1B).

The norditerpenes from *S.* sp. are relatively abundant and novel in structures. Norcembranoids are the most structurally diverse norditerpenes produced by *S.* sp. Compared with cembranoids, norcembranoids are those that lack a C-18 carbon substituent on C-4 [1] and always have a furane heterocycle. They are a rich source of bioactive substances with intriguing and unique structural features (Figure 4). Other types of norditerpenes such as sinulariane-type, horane-type, normandapamatane-type, and sinulanorcembrane-type were also reported in recent years [13,28,71,85].

A new 4-norcembranoidal diterpene possessing a γ-lactone ring, 4α-hydroxy-5-episinuleptolide (**148**) was isolated from a cultured soft coral *S. numerosa* [66]. Cytotoxicity toward CCRF-CEM (human acute lymphoblastic leukemia), HL-60, K-562, U-937 (human histiocytic lymphoma), DLD-1 (human colorectal adenocarcinoma), LNCaP, and MCF7 tumor cells were studied. Compound **148** exhibited moderate cytotoxicity toward CCRF-CEM cells [66].

Two new norcembranoids, sinumerolide A (**149**) and its epimer, 7*E*-sinumerolide A (**150**), were isolated from the soft coral *S. numerosa* [61]. Norcembranoids **149** and **150** were found to inhibit the accumulation of the pro-inflammatory inducible nitric oxide synthase protein of lipopolysaccharide-stimulated RAW264.7 macrophage cells significantly [61].

Four new isoprenoids, including two norcembranoids sinulerectols A and B (**151** and **152**), a cembranoid sinulerectol C (**96**), and a degraded cembranoid sinulerectadione (**153**), were isolated from Dongsha Atoll soft coral *S. erecta* [3]. Compounds **151** and **152** exhibited strong inhibitory effects on superoxide anion generation [3]. They also exhibited potent inhibitory activity against elastase release in the fMLP/CB stimulated cells [3]. Moreover, compounds **151** and **152** were found to show potent activities in the inhibition of superoxide generation and elastase release [3]. Compound **153** exhibited cytotoxicity toward K-562 and MOLT-4 cancer cell lines [3].

One new polycyclic furanobutenolide-derived norcembranoid was isolated from xiguscabrolide H (**154**), from the South China Sea soft corals *S. scabra* [24], and it exhibited potent inhibitory activities on the proliferation of Con A-induced T lymphocyte cells and LPS-induced B lymphocyte cells [24].

A rare sinulariane-type norditerpene sinulariadiolide B (**155**) with a unique cyano group was isolated from the South China Sea soft coral *S. multiflora* [28]. The cytotoxic activities were evaluated against selected cell lines including HL-60, K562, HCT-116, A549, BEL-7402, HeLa, and L-02. All compounds were not cytotoxic toward the above seven cell lines [28]. However, **155** showed weak activity for antifouling against barnacle *B. albicostatus* [28].

A seco-yonarane norditerpenoid, 4,5-secosinulochmodin C (**158**), was isolated from the soft coral *S. inelegans* [58]. Compound **158** was an unprecedented skeleton with an eight-carbon side chain bearing an isopropenyl substituent and terminated by an a-keto ester functionality, which appears to be a C4–C5 redox bond cleavage product of sinulochmodin C [58].

A new horane-type norditerpenoid, named kavaranolide (**159**), was isolated from the Indian soft coral *S. kavarattiensis* [71]. It possessed a tricyclic carbocycle with the trans-fused six and seven-membered rings. The replicon-inhibiting potential of compound **159** was evaluated, but it showed no obvious activity [71].

A new normandapamatane-type norditerpenoid 12-hydroxy-scabrolide A (**160**) and a new norcembranoid 13-*epi*-scabrolide C (**161**) were obtained from the soft coral *S. maxima* [13]. It was confirmed that **161** had little or no effect on cell viability and potently inhibited IL-12 and IL-6 production in LPS-stimulated bone marrow derived dendritic (BMDCs). Compound **161** did not exhibit inhibitory activity on TNF-α production [13].

A new sinulanorcembrane-type norditerpene, 1-*epi*-sinulanorcembranolide A (**162**) was isolated from the soft corals *S. gaweli* [85]. These in vitro anti-inflammatory effects were evaluated, and it did not attenuate the iNOS and COX-2 expression in LPS-stimulated macrophage cells.

A novel tetranorditerpenoid, sinubatin A (**163**) (having an unprecedented carbon skeleton), and a new norditerpenoid, sinubatin B (**164**) (a 4,5-epoxycaryophyllene possessing an unusual methylfuran moiety side chain), were isolated from soft coral *S. nanolobata* [39]. The cytotoxicity of compounds **163** and **164** against mouse lymphocytic leukemia (P-388), human colon adenocarcinoma (HT-29), and human lung epithelial carcinoma (A-549) tumor cell lines were tested but showed no activity [39]. Compounds **163** and **164** were also examined for antiviral activity against human cytomegalovirus (HCMV) and did not show anti-HCMV activity [39].

As shown in Table 4, polycyclic furanobutenolide-derived norditerpenoids were reported to comprise a broad range of biological activities, such as cytotoxic, anti-inflammatory, and immunosuppression and antimalarial activities. Similar to cembranoids, oxygenic rings or hydroxy groups might be critical to the bioactivities. Other types of norditerpenoids showed intriguing structural features with novel carbon skeleton, but their biological activities still need to be explored.

### 2.2. Steroids/Steroidal Glycosides

**Figure 5:**
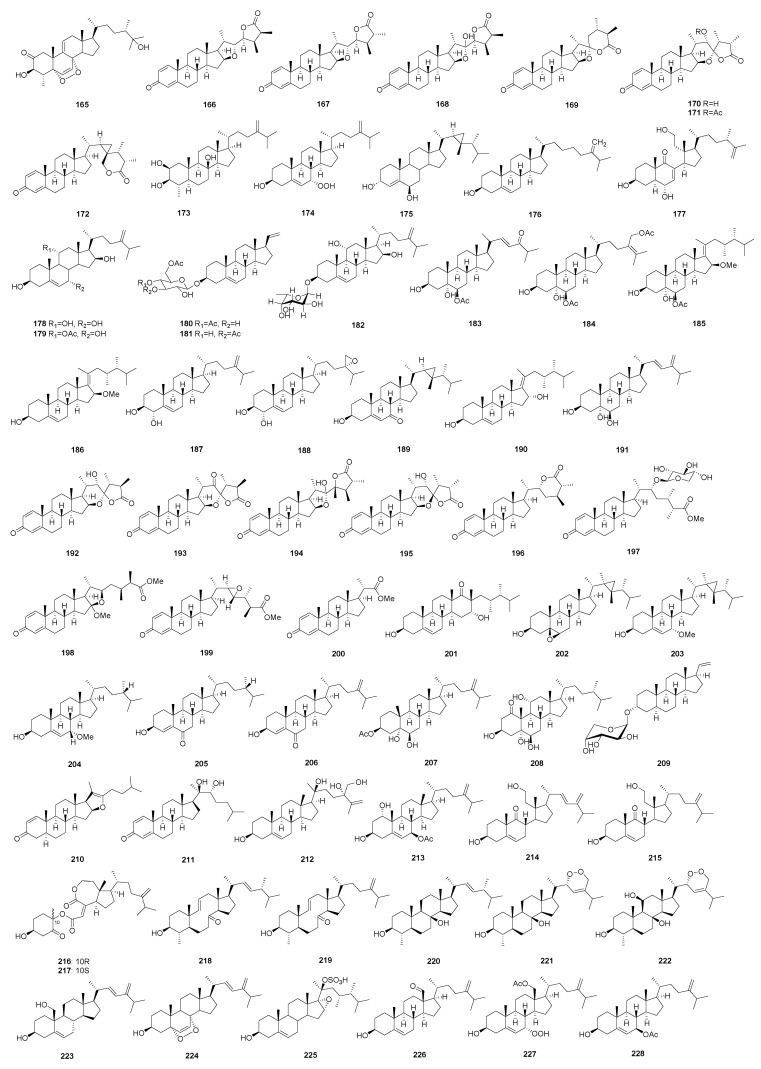
Steroids/steroidal glycosides isolated from genus *Sinularia*.

Among the various classes of secondary metabolites produced by soft corals *S.* sp., steroids come next as a major group of metabolites after terpenoids (Figure 5). The steroid is a kind of natural product with a perhydrocyclopentanophenanthrene skeleton by removing three methyl groups based on lanosterol triterpenoid skeleton. The variations of side chains, high degrees of oxygenation, and re-arrangement on the rings of the tetracyclic nucleus account for the fascinated diversity of such steroids [74]. Among them, withanolides are a group of C_28_ steroidal lactones possessing mostly a C-22/C-26 δ-lactone or in some cases a C-23/C-26 γ-lactone in the side chain [48]. There is also secosteroid produced by *S.* sp. For example, the 9,11-secosteroids are structurally characterized by the C-9/11 oxidative cleavage of the C-ring [25]. Steroids isolated from soft corals also exhibit a broad range of biological activities, such as cytotoxic, anti-inflammatory, anti-microbial, etc.

A novel polyhydroxylated sterol, 3β-25-dihydroxy-4-methyl-5α,8α-epidioxy-2 ketoergost-9-ene (**165**), was isolated from the Red Sea soft coral *S. candidula Safaga* [86]. It showed antiviral activity against the highly pathogenic H5N1 avian influenza strain.

Seven novel withanolides, sinubrasolides A−G (**166**−**172**), have been isolated from the cultured soft coral *S. brassica* [84]. The cytotoxicities of these compounds against the proliferation of cancer cell lines were evaluated, including murine leukemia (P388) cells, lymphoid T carcinoma (MOLT 4) cells, human erythroleukemia (K562) cells, and human colon carcinoma (HT-29) cells. Compound **167** exhibited cytotoxicity toward P388, MOLT 4, and HT-29 cancer cell lines. Compound **170** was found to show cytotoxicity toward MOLT 4 and HT-29 cell lines. In addition, **166** showed cytotoxicity toward the K562 cell line.

Two new steroids, (2β,3β,4α,5α,8β)-4-methylergost-24(28)-ene-2,3,8-triol (**173**) and (3β,7α)-24-methyl-7hydroperoxycholest-5,24(28)-diene-3-ol (**174**) were isolated from the soft coral *S. depressa* Tixier-Durivault [83]. A wide spectrum of biological activity, including neuroprotective, PTP1B inhibitory, and antibacterial properties were tested but did not show obvious activity [83].

A new steroid, dissesterol (**175**), was separated from a Vietnamese soft coral *S. dissecta* [82]. It was confirmed that **175** had no or little effect on the cell viability, and it showed strong suppression of LPS-stimulated IL-12 p40 production [82].

Hurgadacin (**176**), a 24,25-bishomo-26-methylenecholesterol, was isolated from the Red Sea soft coral *S. polydactyla* [80]. It was the first steroids with an extended side chain with two methylene units. Compound **176** displayed only marginal cytotoxicity against brine shrimps [80].

A new 9,11-secosteroid, 25(26)-dehydrosarcomilasterol (**177**), and two new polyhydroxylated steroids, 7α-hydroxy-crassarosterol A (**178**) and 11-acetoxy-7α-hydroxy-crassarosterol A (**179**), were isolated from the South China Sea soft corals *Sarcophyton trocheliophorum* and *S. flexibilis*, respectively [74]. Compound **178** exhibited a moderate protein tyrosine phosphatase 1B (PTP1B) inhibitory activity. Compounds **177**–**179** showed weak in vitro cytotoxicities against the tumor cell lines K562 and HL-60 [74].

Two new pregnane glycosides, hirsutosterosides A and B (**180** and **181**), and a new steroidal glycoside, lobatasteroside A (**182**), were isolated from soft coral *Cladiella hirsute* and *S. nanolobata*, respectively [67]. Compounds **180** and **181** were found to exhibit the corresponding cytotoxicity toward the four cancer cell lines (K562, P388, HT-29, and A549). Moreover, compound **180** significantly inhibited the fMLP/CB-induced elastase release [67].

Four new polyhydroxylated steroids (**183**–**186**) were isolated from the soft coral *S. acuta* collected from Weizhou Island of Guangxi Province, China [60]. Compound **184** showed potent cytotoxicity against HL-60 cell lines. Compounds **183** and **184** showed weak activities against HeLa cell lines [60].

Two new steroids, 3β,4α-dihydroxyergosta-5,24(28)-diene (**187**) and 24(S),28-epoxy-ergost-5-ene-3β,4α-diol (**188**), were isolated from the Vietnamese soft coral *S. nanolobata* [55]. Compound **188** exhibited moderate cytotoxicity against the HL-60 cell line and a weak effect on HepG2 and colon adenocarcinoma (SW480) cell lines [55].

A new polyhydroxysterol (22*E*)-24-methylenecholestane-22-ene-3β,5α,6β-triol (**191**) was isolated from the South China Sea soft coral *S.* sp. [50]. It exhibited a cytotoxic effect against both HepG2 and HeLa cell lines [50].

Five new withanolides sinubrasolides H–L (**192**–**196**) were separated from soft coral *S. brassica* [48]. The results of the cytotoxicity test showed that compounds with a hydroxy group at C-22 (**192**, **194**, and **195**) exhibited cytotoxic activity against P388, MOLT-4, K-562, and HT-29 cell lines, whereas **193** and **196** showed no activity. Compounds **194**–**196** also exhibited moderate inhibitory activities against superoxide anion generation. Compounds **192** and **196** also exhibited moderate inhibitory activities against elastase release [48].

Four new steroids with methyl ester groups, sinubrasones A–D (**197**–**200**), were obtained from a reef soft coral *S. brassica*, which was cultured in a tank [12]. Compound **197** possesses a β-D-xylopyranose. Compounds **198** and **199** were found to show significant cytotoxicity against P388D1, MOLT-4, K-562, and HT-29 cell lines. Compounds **197** and **200** exhibited only weak cytotoxic activity against P388D1 and MOLT-4 cell lines. Moreover, **200** showed a significant inhibitory effect (53.6 ± 1.8%) against superoxide anion generation. Compounds **199** and **200** also exhibited inhibitory activities against elastase release [12].

Two new cytotoxic compounds, leptosteroid (**201**) and 5,6β-epoxygorgosterol (**202**), were isolated from the Vietnamese soft coral *S. leptoclados* [46]. Compound **201** showed significant cytotoxicity against hepatoma cancer and colon adenocarcinoma cell lines [46].

Five new steroids (**203**–**207**) were isolated and structurally elucidated from a methanol extract of the Vietnamese soft coral *S. conferta* [45]. Their cytotoxic effects against three human cancer cell lines, lung carcinoma (A-549), cervical adenocarcinoma (HeLa), and pancreatic epithelioid carcinoma (PANC-1), were evaluated. However, none of them showed obvious activities [45].

A new ergostane-type steroid sinubrassione (**208**) and a new pregnene-type steroid glycoside sinubrassioside (**209**) were isolated from methanol extract of the Vietnamese soft coral *S. brassica* [43]. Cytotoxic activity was observed for compound **208** against PANC-1. No cytotoxicity was observed for compound **209**, suggesting that the location of the sugar moiety may be important for the cytotoxicity [43].

Two new steroids, ximaosteroid E (**210**) and ximaosteroid F (**211**), were separated from the Chinese soft coral *S.* sp. [11]. Compound **210** possessed an uncommon dihydrofuran group. Compounds **210** and **211** showed significant cytotoxicity against the HL-60 tumor cell line [11].

Two new highly oxygenated ergostane-type sterols (**212** and **213**) were isolated from the soft coral *S.* sp. collected from the Xisha Islands, South China Sea [9]. Compounds **212** and **213** exhibited moderate anti-proliferation effects against five human cancer cell lines, including MDA-MB-436, A549, Hep3B, HT-29, and H157. Compound **212**-treated H157 cells displayed apoptosis characteristics. Moreover, Western blot assays suggested that **212** could increase the expression of Bax and down-regulate the expression of Bcl-2 [9].

Two novel 9,11-secosteroids named sinleptosterols A (**214**) and B (**215**) were discovered from Taiwan soft coral *S. leptoclados* [25]. Compounds **214** and **215** were shown to inhibit superoxide anion generation and elastase release by human neutrophils in response to fMLP/CB [25].

A pair of novel highly degraded steroid named Erectsterates A and B (**216** and **217**) were derived from the South China Sea soft coral *S. erecta* [2]. These two compounds are rare steroids with a high degradation in ring B and an ester linkage between the A and C/D rings. It was proposed that the ring C of **216** and **217** was formally oxidized by the Baeyer–Villiger reaction to provide an unprecedented seven-membered lactone moiety in ring C of steroid [2]. Compound **217** showed a weak inhibitory effect on the A549, HT29, SNU-398 and Capan-1 cell lines [2].

Six new steroids (**218**–**223**) were isolated from the organic extract of the soft coral *S. polydactyla* collected from the Hurghada reef in the Red Sea [6]. Compounds **218** and **219** displayed the rare 8,9-seco-cholestane steroidal nucleus. Compound **223** displayed increased inhibition of androgen receptors with decreasing concentrations [6].

A new 5α,8α-epidioxysterol yalongsterol A (**224**) was isolated from the South China Sea soft coral *S.* sp. [22]. Compound **224** exhibited moderate activities against the concanavalin A (ConA)-induced proliferation of T lymphocyte cells and LPS-induced proliferation of B lymphocyte cells [22].

A new steroid named 16,17-epoxy-23-methylergostane (**225**) was derived from *S. variabilis*, which is a soft coral from the Persian Gulf [19]. It showed cytotoxic activity in a dose-dependent reduction manner against MCF-7 and MDA-MB-231. Apoptosis was the underlying mechanism that was enrolled by **225** to induce cell death [19].

Three new oxygenated steroids, sinulasterols A–C (**226**–**228**), were isolated from the Chinese soft coral *S. depressa* [5]. Compounds **226** and **228** featured unusual C-18 oxygenated patterns. In the TNF-α bioassay, **226** and **227** displayed a moderate inhibitory activity [5].

The biological activities of new steroids from *S.* sp. are summarized in Table 5. Many steroids showed interesting cytotoxicity to cancer cell lines, especially withanolides, with a C-22/C-26 δ-lactone or a C-23/C-26 γ-lactone, which showed significant bioactivities. Some of them displayed anti-inflammatory activity. It is worth mentioning that hydroxy or oxygenic rings on the side chain might be a critical point for the activities, in particular, the cytotoxicities.

### 2.3. Other Types

**Figure 6:**
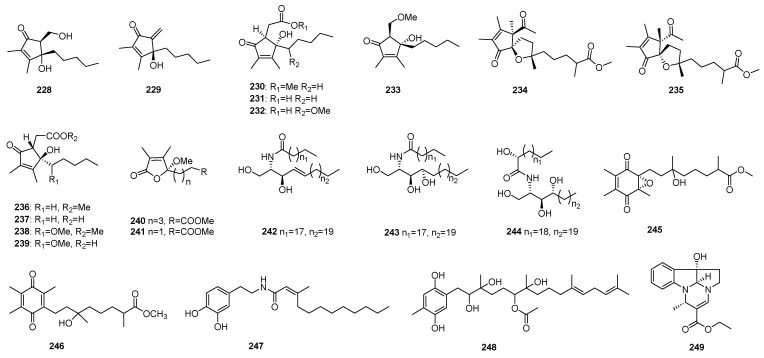
Other types of structures isolated from genus *Sinularia*.

In addition to a rich harvest of terpenoids and steroids/steroidal glycosides, *S.* sp. also yields other kinds of metabolites, such as cyclopentenone, ceramides, alkaloid, quinones, and lipids (Figure 6).

Two new cyclopentenone derivatives, (4S*,5S*)-4-hydroxy-5-(hydroxymethyl)-2,3-dimethyl-4-pentylcyclopent-2-en-1-one (**228**) and (*S*)-4-hydroxy-5-methylene-2,3-dimethyl-4-pentylcyclopent-2-en-1-one (**229**), were isolated from a South China Sea soft coral *S. verruca* [49]. Compounds **228** and **229** were found to be moderately protective against the cytopathic effects of in vitro HIV-1 infection with EC_50_ values of 34 and 5.8 μM, respectively, and maximum protection rates of 86% and 52%, respectively. Their IC_50_ values for cytotoxicity against the CEM-SS host cells were determined to be 79 and 6.3 μM. Moreover, compound **228** showed moderate inhibition against lipopolysaccharide-induced NO production in mouse peritoneal macrophages with IC_50_ values of 28 µM [49].

Three new cyclopentenone derivatives, ent-sinulolides C, D, and F (**230**–**232**) were isolated from the Paracel Islands soft coral *S.* cf. *molesta* [40]. Their cytotoxic (against HeLa, HCT-116, BEL-7402, K562, and Jurkat) and inhibitory activities against protein tyrosine phosphatase 1B (PTP1B) were evaluated but showed no obvious activity [40].

Eight new cyclopentenone and butenolide derivatives, sinulolides A–H (**234**–**241**), were isolated from the soft coral *S.* sp. [68]. At a concentration of 10 μg/mL, sinulolide E exhibited moderate effects with an inhibitory rate of 38.12% [68].

Three new ceramides, *N*-[(2*S*,3*R*,*E*)-1,3-dihydroxyhexacos-4-en-2-yl]icosanamide (**242**), *N*-[(2*S*,3*S*,4*R*)-1,3,4-trihydroxyhexacosan-2-yl]icosanamide (**243**), and (*R*)-2′-hydroxy-*N*-[(2*S*,3*S*,4*R*)-1,3,4-trihydroxypentacosan-2-yl] nonadecanamide (**244**), were isolated from the Red Sea soft coral *S. candidula* [86]. The compounds **242**–**244** showed a reduction of virus titer by 48.81%, 10.43%, and 15.76% at a concentration of 1 ng/mL, respectively.

A new quinone derivative, flexibilisquinone (**246**), was isolated from the cultured soft coral *S. flexibilis* [92]. In the in vitro anti-inflammatory effects test, quinone **246** was found to significantly inhibit the accumulation of the pro-inflammatory iNOS and COX-2 proteins of the LPS-stimulated RAW264.7 macrophage cells. Both iNOS and COX-2 were significantly inhibited by compound **246** at 5–20 µM and 20 µM, respectively. Furthermore, compound **246** (1–20 µM) did not induce obvious cytotoxicity in macrophage cells.

## 3. Conclusions

Marine invertebrates have been regarded as a treasurable source of bioactive secondary metabolites for drug development. Coral reefs are among the most productive marine ecosystems, which produced a huge diversity of chemical structures with biological properties. The soft corals belonging to the family *Sinularia* are not an exception. An increasing number of compounds have been reported since the first report of this genus published in 1975 [93]. With the rapid development of analysis, separation, and structure identification technology, more and more new compounds have been isolated and identified each year from soft coral *S.* sp. Until now, more than 150 species of this genus have been found, 78 species among which have been chemically studied, and more than 700 metabolites have been reported. In this review, we have reported 249 compounds along with different structures and potential biological activity based on the data collected from the available literature. The chemical diversity of the structures can be attributed to the chemical groups and the arrangement of the core structure. In particular, sulfur or nitrogen-containing metabolites are a special, relatively rare, and important class of natural products from soft coral (such as compounds **59** and **249**), which suggested that the symbiotic microorganisms are the real producers. According to the reported literature, soft coral also harbors a microbial community featuring sponges and ascidians [94], including fungus, bacteria, and actinobacteria [95,96,97,98], which also corresponds to the antimicrobial activity of coral-derived natural products. The extensive structure–activity relationship studies on the metabolites from *Sinularia* species are expected, which provide a direction for the discovery of lead compounds. There are also some compounds with novel structures including some new skeletons that tested negative for the limited activity test models and low yield. Therefore, the structure-based drug design methods [18] (e.g., molecular docking, structure-based virtual screening and molecular dynamics) can be utilized in accordance with activity screening.

## Figures and Tables

**Table 1:** Chemically studied *Sinularia* soft coral from 2013 to March 2021.

Species	Sampling Locations	Authors	Ref.
*S. humilis*	Ximao Island, Hainan Province, China	Li-Li Sun; Xu-Wen Li *	[4]
*S. variabilis*	the Persian Gulf	Pardis Mohammadi Pour; Mustafa Ghanadian *	[19]
*S. scabra*	Pingtung, southern Taiwan	Yu-Hung Lu; Mohamed El-Shazly *	[20]
*S. polydactyla*	Xisha Islands, Hainan Province, China	Fei Ye; Xu-wen Li *	[21]
*S.* sp.	Yalong bay, Sanya, China	Min Yang; Yue-Wei Guo *	[22]
*S. depressa*	Ximao Island, Hainan Province, China	Min Yang; Yue-Wei Guo *	[5]
*S. polydactyla*	Hurghada, Egypt	Mohamed A. Tammam; Vassilios Roussis *	[6]
*S. erecta*	Ximao Island, Hainan Province, China	Jiao Liu; Yue-Wei Guo *	[2]
*S. erecta*	Ximao Island, Hainan Province, China	Jiao Liu; Yue-Wei Guo *	[7]
*S. humilis*	Ximao Island, Hainan Province, China	Jie Li; Xu-wen Li *	[23]
*S. scabra*	Xigu Island, Hainan Province, China	Wan-Xiang Cui; Yue-Wei Guo *	[24]
*S. polydactyla*	Ximao Island, Hainan Province, China	Wan-Xiang Cui; Yue-Wei Guo *	[24]
*S. leptoclados*	Pingtung, Taiwan	Yu-Chia Chang; Tsong-Long Hwang *	[25]
*S. tumulosa*	Ximao Island, Hainan Province, China	You-Sheng Cai; Yue-Wei Guo *	[8]
*S. scabra*	Grand Island, NY, USA	Min Yang; Xu-wen Li *	[26]
*S. flexibilis*	Xidao Island, Hainan	Qihao Wu; Yue-Wei Guo *	[27]
*S. multiflora*	Xisha Islands of the South China Sea	Zheng Wang; Guo-Qiang Li *	[28]
*S. flexibilis*	Mantanani Island, Sabah, Malaysia	Chin-Soon Phan; Takashi Kamada *	[29]
*S. hirta*	Yalong Bay, Hainan, China	Si-Qi Lu; Guan-Ying Han *	[30]
*S.* sp.	Zhanjiang, Guangdong Province, China	Wei Lai; Shi-Hai Xu *	[31]
*S.* sp.	Xisha Island, South China Sea, China	Cheng-Shi Jiang; Yue-Wei Guo *	[32]
*S.* sp.	Xisha Island, South China Sea, China	Cheng-Shi Jiang; Yue-Wei Guo *	[9]
*S. digitata*	Tho Chu island, Phu Quoc, Kien Giang, Vietnam	Nguyen Thi Huong; Nguyen Hoai Nam *	[33]
*S. polydactyla*	Xisha Island, South China Sea, China	Fei Ye; Yue-Wei Guo *	[34]
*S. flexibilis*	Liuqiu, Taiwan	Chia-Hua Wu; Jyh-Horng Sheu *	[35]
*S.* sp.	Yongxing Island, Xisha Islands, the South China Sea	Guo-Fei Qin; Guo-Qiang Li *	[10]
*S.* sp.	Ximao Island, Hainan Province, China	Song-Wei Li; Yue-Wei Guo *	[11]
*S.* sp.	Mantanani Island, Sabah	Takashi Kamada; Charles S. Vairappan *	[36]
*S. flexibilis*	Mengalum Island, Sabah	Takashi Kamada; Charles S. Vairappan *	[37]
*S. erecta*	Cu Lao Cham island, Quang Nam, Vietnam	Nguyen Thi Huong; Chau Van Minh *	[38]
*S. nanolobata*	San-Shin-Tai, Taitong County, Taiwan	Fu-Yun Hsu; Chang-Yih Duh *	[39]
*S.* cf. *molesta*	the Paracel Islands, the South China Sea	Mei-Jun Chu; Guo-Qiang Li *	[40]
*S. polydactyla*	Xisha Island, the South China Sea	Fei Ye; Yue-Wei Guo *	[41]
*S. flexibilis*	Sanya Bay, Hainan Island, China	Jia Wang; Sheng An Tang *	[42]
*S. brassica*	Con Co Island, Quangtri province, Vietnam	Hong Hanh Thi Tran; Chau Van Minh *	[43]
*S. nanolobata*	Lang Co, Hue, Vietnam	Ninh Thi Ngoc; Chau Van Minh *	[44]
*S. conferta*	Con Co island, Quangtri, Vietnam	Ninh Thi Ngoc; Chau Van Minh *	[45]
*S. leptoclados*	Con Co island, Quangtri, Vietnam	Ninh Thi Ngoc; Chau Van Minh *	[46]
*S. terspilli*	Hurghada, Egypt	Rabab Mohammed; Mahmoud A. ElSohly *	[47]
*S. brassica*	Cultured, Taiwan	Chiung-Yao Huang; Jyh-Horng Sheu *	[12]
*S. brassica*	Cultured, Taiwan	Chiung-Yao Huang; Jyh-Horng Sheu *	[48]
*S. verruca*	Ximao Island, Hainan Province, China	Weiping Yuan; Pengcheng Yan *	[49]
*S.* sp.	Zhanjiang, Guangdong Province, China	Huan Sun; Shi-Hai Xu *	[50]
*S.* sp.	Irabu Island, Okinawa, Japan	Prodip K. Roy; Katsuhiro Ueda *	[51]
*S. vanderlandi*	Madagascan	Rahelivao M.; Hans-Joachim Knölker *	[52]
*S.* sp.	Mantanani Island, Sabah, Malaysia	Chin-Soon Phan; Charles S. Vairappan *	[53]
*S. microspiculata*	Da Den, Quangninh, Vietnam,	Nguyen Van Thanh; Chau Van Minh *	[54]
*S. nanolobata*	Lang Co., Hue, Vietnam	Ninh Thi Ngoc; Chau Van Minh *	[55]
*S. lochmodes*	Northeast corner of Taiwan	Yun-Sheng Lin; Jyh-Horng Sheu *	[56]
*S. erecta*	Dongsha Atoll	Chiung-Yao Huang; Jyh-Horng Sheu *	[3]
*S. polydactyla*	Red Sea, Hurghada, Egyptian	Mohamed-Elamir F. Hegazy; Paul W. Paré *	[57]
*S. inelegans*	Mandapam, the Gulfof Mannar, India	Bhujanga Rao Chitturi *	[58]
*S. nanolobata*	Jihui Fishing Port, Taitung County, Taiwan	Chih-Hua Chao; Jyh-Horng Sheu *	[59]
*S. acuta*	Weizhou Island, Guangxi Province, China	Nai-Xia Zhang; Guo-Qiang Li *	[60]
*S. numerosa*	Cultured, Taiwan	Chen-Ting Yin; Ping-Jyun Sung *	[61]
*S.* sp.	Dongluo Island, Hainan Province, China	Bin Yang; Yonghong Liu *	[62]
*S. flexibilis*	Cultured, Taiwan	Tsung-Chang Tsai; Jui-Hsin Su *	[63]
*S. gaweli*	Sansiantai, Taitung County, Taiwan	Wun-Jie Lin; Ping-Jyun Sung *	[64]
*S. flexibilis*	Yalong Bay, Hainan Province, China,	Wen-Ting Chen; Yue-Wei Guo *	[65]
*S. numerosa*	Cultured, Taiwan	Wu-Fu Chen; Ping-Jyun Sung *	[66]
*S. nanolobata*	Sianglu Islet, Penghu Islands, Taiwan	Chih-Hua Chao; Jyh-Horng Sheu *	[67]
*S.* sp.	Dongluo Island, Hainan Province, China	Bin Yang; Yonghong Liu *	[68]
*S. arborea*	Taiwan	Li-Hsueh Wang; Ping-Jyun Sung *	[69]
*S. kavarattiensis*	Mandapam, Tamil Nadu, India	S Rajaram; Y Venkateswarlu *	[70]
*S. kavarattiensis*	Rameshwaram, Tamil Nadu, India	Katja-Emilia Lillsunde; Päivi Tammela *	[71]
*S.* sp.	Sanya Bay, Hainan Island, China	Ling-Fang Lei; Cui-Xian Zhang *	[72]
*S. depressa*	Lingshui Bay, Hainan Province, China	Cheng-shi Jiang; Yue-Wei Guo *	[73]
*S. flexibilis*	Yalong Bay, Hainan Province, China	Wen-Ting Chen; Yue-Wei Guo *	[74]
*S. arborea*	Taiwan	Kuan-Hua Chen; Ping-Jyun Sung *	[75]
*S. capillosa*	Sanya Bay, Hainan Island, China	Dawei Chen; Wenhan Lin *	[76]
*S.* sp.	Ximao island, Hainan Province, China	Jian Yin; Pengcheng Yan *	[77]
*S.* sp.	Dongluo Island, Hainan province, China	Bin Yang; Yonghong Liu *	[78]
*S. leptoclados*	Cultured, Taiwan	Tsung-Chang Tsai; Yun-Sheng Lin *	[79]
*S. maxima*	Nha Trang Bay, Vietnam	Nguyen Phuong Thao; Young Ho Kim *	[13]
*S. polydactyla*	Red Sea, Hurghada, Egyptian	Mohamed Shaaban *	[80]
*S. kavarattiensis*	Mandapam, Tamilnadu, India	Singanaboina Rajaram; Suryakiran Navath *	[81]
*S. dissecta*	Hai Van-Son Cha, Hue, Vietnam	Nguyen Phuong Thao; Young Ho Kim *	[82]
*S. depressa*	Lingshui Bay, Hainan, China	Lin-Fu Liang; Yue-Wei Guo *	[83]
*S. rigida*	Sanya Bay, Hainan Island of China	Daowan Lai; Wenhan Lin *	[14]
*S. brassica*	Cultured, Taiwan	Chiung-Yao Huang; Jyh-Horng Sheu *	[84]
*S. flexibilis*	Sansiantai, Taitung County, Taiwan	Li-Chung Hu; Ping-Jyun Sung *	[85]
*S. candidula*	Egyptian Red Sea	Safwat Ahmed *	[86]
*S. polydactyla*	the Red Sea	El-Sayed A. Aboutabl; Ahmed A. Hussein *	[87]
*S. crassa*	West Island, the South China Sea	Meng-Jun Wu; Yue-Wei Guo *	[88]

^*^ corresponding author.

**Table 2:** Biological activities of new sesquiterpenes from genus *Sinularia*.

Drug Class	Compounds	Pharmacology	Activities	Ref.
Anti-inflammatory	sinularianins C–F (**1**–**4**)	NF-κB inhibition	inhibition rate: 24.3–43.0%, 10 µg/mL	[78]
	capillosananes W (**9**)	TNF-α inhibition	inhibition rate: 34%, 10 µM	[76]
	sinulatumolin A (**32**)	TNF-α inhibition	IC_50_: 7.5 μM	[8]
	sinulatumolin C (**33**)	TNF-α inhibition	IC_50_: 2.6 μM	[8]
	sinulatumolin D (**34**)	TNF-α inhibition	IC_50_: 3.6 μM	[8]
Antibacterial	**13**	against *Staphylococcus aureus*	MIC: 18.75 µg/mL	[70]
	**16**–**18**	against *Staphylococcus aureus* and *Salmonella enteric*	<12 mm ^a^, 25 µg/disc	[51]
	**17**, **18**	against *Serratia marcescens*	<14 mm ^a^, 26 µg/disc	[51]
Cytotoxicity	**16**–**18**	against HCT 116	IC_50_: 43.6–75.34 μM	[51]
	**27**	against A549	IC_50_: 14.79 ± 0.91 μM	[38]
	sinuketal (**28**)	against Jurkat, MDA-MB-231, U2OS	IC_50_: 24.9, 32.3, 41.7 µM	[10]
Antimalarial	sinuketal (**28**)	against *Plasmodium falciparum* 3D7	IC_50_: 80 µM	[10]
Antidiabetic	molestin C (**26**)	PTP1B inhibition	IC_50_: 218 µM	[40]

^a^ inhibition zone.

**Table 3:** Biological activities of new diterpenes from genus *Sinularia*.

Drug Class	Compounds	Pharmacology	Activities	Ref
Anti-inflammatory	sinularcasbane B (**51**)	inhibit NO production	IC_50_: 8.3 μM	[77]
	sinularcasbane E (**54**)	inhibit NO production	IC_50_: 5.4 μM	[77]
	nanoculone B (**86**)	inhibit NO production	inhibition rate: 8%, 10 μM	[59]
	sinularolide F (**104**)	inhibit NO production	IC_50_ < 6.25 µg/mL	[36]
	humilisin F (**139**)	inhibit NO production	inhibition rate: 83.96%, 10 μM; 65.70%, 20 μM	[4]
	arbolide C (**63**)	inhibit release of elastase	IC_50_: 5.13 μg/mL	[69]
	sinulerectol C (**96**)	inhibit release of elastase	inhibition rate: 33%, 10 μM	[3]
	sinularbol B (**57**)	inhibit superoxide anion generation	inhibition rate 23.94%, 10 μg/mL	[75]
	flexibilin D (**36**)	iNOS and COX-2 inhibition	inhibition rate: 19.27% and 30.08%, 20 μM	[85]
	isosinulaflexiolide K (**78**)	iNOS and COX-2 inhibition	inhibition rate: 30.9% and 47.1%, 10 μM	[63]
	sinulacembranolide A (**73**)	iNOS inhibition	inhibition rate: 8.55%, 10 μM	[64]
	xidaosinularide A (**118**)	TNF-α inhibition	IC_50_: 38.9 μM	[27]
	sinueretone A (**128**)	TNF-α inhibition	inhibition rate: 21.9%, 20 μM	[7]
	sinuereperoxide A (**129**)	TNF-α inhibition	inhibition rate: 56.2%, 20 μM, IC_50_: 10.6 μM	[7]
Cytotoxicity	leptoclalin A (**49**)	against T-47D and K-562	IC_50:_ 15.4 and 12.8 μg/mL	[79]
	sinulariaoid A (**59**)	against HepG2, HepG2/ADM, MCF-7, and MCF-7/ADM	IC_50_: 15.35, 9.70, 18.41 and 16.95 μM	[72]
	sinulerectol C (**96**)	against K-562	IC_50_: 9.2 μM	[3]
	pambanolides B–C (**91** and **92**)	against DU145 and A549	IC_50_: 63–55 mM	[58]
	xishacorenes A−C (**99**–**101**)	promote the ConA-induced T lymphocytes proliferation	dose-dependency (10–40 μM)	[41]
	molestin E (**102**)	against HeLa and HCT-116	IC_50:_ 5.26 and 8.37 μM	[40]
	*ent*-sinuflexibilin D (**103**)	against S1T	IC_50_: 5.27 μg/mL	[37]
	sinularolide F (**104**)	against HL-60	cell viability: <30%, 25.0 µg/mL	[36]
Antimalarial	sinulariol Z_5_ (**48**)	against barnacle *Ba. amphitrite*	EC_50_: 4.57 μg/mL	[14]
		against *Bu. neritina*	EC_50_: 13.48 μg/mL	[14]
	multifloralin (**144**)	barnacle *Balanus albicostatus*	adhesive rate: 0%; lethal rate: 58.11%, at 25 ppm	[28]
Antidiabetic	sinupol (**111**)	PTP1B inhibition	IC_50_: 63.9 µM	[34]
	sinulacetate (**112**)	PTP1B inhibition	IC_50_: 51.8 µM	[34]
	sinulacrassin (**141**)	α-glucosidase inhibitor	IC_50_: 10.65 µM	[88]
Antibacterial	prenyl-α-elemenone (**97**)	against *S. aureus*	MBC: 50 µg mL^−1^; MIC: 20 µg mL^−1^	[53]
Antifungal	sinulaflexiolide P (**117**)	against *H. milfordensis*	MIC: 25 μg/mL	[29]
Anti-AD	**113** and **115**	inhibit Aβ_42_ aggregation	inhibition rate: 20.6% and 37.2%, respectively, 10 μM	[32]
Immunosuppression	xiguscabrate B (**122**)	inhibit Con A-induced T lymphocyte cells proliferation	IC_50_: 8.4 μM	[26]
	xiguscabrol A (**124**)	inhibit Con A-induced T lymphocyte cells proliferation	IC_50_: 5.5 μM	[26]
	xiguscabrol B (**125**)	inhibit Con A-induced T lymphocyte cells proliferation	IC_50_: 3.9 μM	[26]
	8-*epi*-xiguscabrol B (**126**)	inhibit Con A-induced T lymphocyte cells proliferation	IC_50_: 2.3 μM	[26]

**Table 4:** Biological activities of new norterpenoids from genus *Sinularia*.

Drug Class	Compounds	Pharmacology	Activities	Ref
Anti-inflammatory	sinumerolide A (**149**)	inhibit NO production	inhibition rate: 59.82%, 10 µM	[61]
	7*E*-sinumerolide A (**150**)	inhibit NO production	inhibition rate: 68.40%, 10 µM	[61]
	sinulerectol A (**151**)	inhibit superoxide anion generation and release of elastase	IC_50_: 2.3, 0.9 μM	[3]
	sinulerectol B (**152**)	inhibit superoxide anion generation and release of elastase	IC_50_: 8.5, 3.8 μM	[3]
	13-epi-scabrolide C (**161**)	inhibit IL-12 and IL-6	IC_50_: 5.3, 13.12 μM	[13]
Cytotoxicity	4α-hydroxy-5-episinuleptolide (**148**)	against CCRF-CEM	IC_50:_ 4.21 μg/mL	[40]
	sinulerectadione (**153**)	against K-562 and MOLT-4	IC_50_: 8.6, 9.7 μM	[3]
Antidiabetic	molestins D (**147**)	PTP1B inhibition	IC_50_: 344 μM	[40]
Immunosuppression	xiguscabrolide H (**154**)	inhibit T and B lymphocyte cells proliferation	IC_50_: 45.76, 44.14 μM	[3]
Antimalarial	sinulariadiolide B (**155**)	against barnacle *B. albicostatus*	adhesive rate: 41.55%, at 25 ppm	[24]

**Table 5:** Biological activities of new steroids from genus *Sinularia*.

Drug Class	Compounds	Pharmacology	Activities	Ref
Cytotoxicity	sinubrasolide B (**167**)	against P388, MOLT 4 and HT-29	ED_50_: 9.1, 4.8, 4.8 μM	[84]
	sinubrasolide E (**171**)	against K562	ED_50_: 9.9, 7.5 μM	[84]
	sinubrasolide A (**166**)	against MOLT 4 and HT-30	ED_50_: 8.7, 7.6 μM	[84]
	**177**–**179**	against K562 and HL-60	IC_50_: 36.28–93.43 μM	[74]
	**183**	against HeLa	IC_50_: 44.8 μM	[60]
	**184**	against HL-60 and HeLa	IC_50_: 7.3, 27.1 μM	[60]
	**188**	against HL-60, HepG2, SW480	IC_50_: 33.53, 64.35, 71.02 μM	[55]
	**191**	against HepG2 and HeLa	IC_50_: 37.30, 19.32 μM	[50]
	sinubrasolide H (**192**)	against P388, MOLT-4, K-562 and HT-29	IC_50_: 39.8, 28.6, 29.7, 24.4 μM	[48]
	sinubrasolide J (**194**)	against P388, MOLT-4, K-562 and HT-30	IC_50_: 18.7, 17.2, 12.6, 11.2 μM	[48]
	sinubrasolide K (**195**)	against P388, MOLT-4, K-562 and HT-31	IC_50_: 18.3, 13.7, 17.4, 20.5 μM	[48]
	sinubrasone A (**197**)	against P388D1, MOLT-4	IC_50_: 37.2, 37.8 μM	[48]
	sinubrasone B (**198**)	against P388D1, MOLT-4, K-562, and HT-29	IC_50_: 9.7, 6.0, 5.2, 7.6 μM	[12]
	sinubrasone C (**199**)	against P388D1, MOLT-4, K-562, and HT-29	IC_50_: 5.7, 5.3, 12.1, 10.4 μM	[12]
	sinubrasone D (**200**)	against P388D1, MOLT-4, K-562, and HT-29	IC_50_: 24.4, 31.2, 21.3, 36.5 μM	[12]
	leptosteroid (**201**)	against HepG2 and SW480	IC_50_: 21.13, 28.65 μM	[46]
	sinubrassione (**208**)	against PANC-1	IC_50_: 15.24 μM	[43]
	ximaosteroid E (**210**)	against HL-60	IC_50_: 1.79 μM	[11]
	ximaosteroid F (**211**)	against HL-60	IC_50_: 4.03 μM	[11]
	**212**	against MDA-MB-436, Hep3B, HT-29, and H157	IC_50_: 17.15, 29.28, 30.06, 10.14 μM	[9]
	**213**	against MDA-MB-436, A549, Hep3B, HT-29	IC_50_: 18.21, 41.71, 19.03, 10.38 μM	[9]
	erectsterate (**217**)	against A549, HT29, SNU-398 and Capan-1	IC_50_: 40.55, 32.83, 15.57, 23.51 μM	[2]
	16,17-epoxy-23-methylergostane (**225**)	against MCF-7 and MDA-MB-231	IC_50_: 31.44, 25.67 μM	[19]
	**223**	inhibit androgen receptors	inhibition rate: >100%, 10 μM	[6]
Anti-inflammatory	dissesterol (**175**)	IL-12 p40 inhibition	IC_50_: 4.0 μM	[82]
	sinubrasolide H (**192**)	inhibit release of elastase	inhibition rate: 32.4%, 10 μM	[48]
	sinubrasolide J (**194**)	inhibit superoxide anion generation	inhibition rate: 32.1%, 10 μM	[48]
	sinubrasolide K (**195**)	inhibit superoxide anion generation	inhibition rate: 34.3%, 10 μM	[48]
	sinubrasolide L (**196**)	inhibit superoxide anion generation and release of elastase	inhibition rate: 26.3%, 25.0%, 10 μM	[48]
	sinubrasone C (**199**)	inhibit the release of elastase	inhibition rate: 58.8%, 10 μM	[12]
	sinubrasone D (**200**)	inhibit the release of elastase	inhibition rate: 66.3%, 10 μM	[12]
	sinleptosterol A (**214**)	inhibit superoxide anion generation and release of elastase	IC_50_: 7.07, 7.57 μM	[25]
	sinleptosterol B (**215**)	inhibit superoxide anion generation and release of elastase	IC_50_: 4.68, 4.29 μM	[25]
	sinulasterol A (**226**)	TNF-α inhibition	IC_50_: 51.1 μM	[5]
	sinulasterol B (**227**)	TNF-α inhibition	IC_50_: 22.7 μM	[5]
Antidiabetic	7α-hydroxy-crassarosterol A (**178**)	PTP1B inhibition	IC_50_: 33.05 μM	[74]
Immunosuppression	yalongsterol A (**224**)	inhibit T and B lymphocyte cells proliferation	IC_50_: 46.0, 56.5 μM	[22]
Antiviral	**165**	against H5N1	inhibition rate: 55.16%, 1 ng/mL	[84]

## Data Availability

The data presented in this study are available.

## References

[1] Chen W.-T., Li Y., Guo Y.-W. (2012). Terpenoids of *Sinularia* soft corals: Chemistry and bioactivity. Acta Pharm. Sin. B.

[2] Liu J., Yang M., Miao Z.-H., Wu X., Gu Y.-C., Yao L.-G., Huan X.-J., Luo H., Guo Y.-W. (2020). Erectsterates A and B, a pair of novel highly degraded steroid derivatives from the South China Sea soft coral *Sinularia erecta*. Steroids.

[3] Huang C.-Y., Tseng Y.-J., Chokkalingam U., Hwang T.-L., Hsu C.-H., Dai C.-F., Sung P.-J., Sheu J.-H. (2016). Bioactive isoprenoid-derived natural products from a Dongsha Atoll soft coral *Sinularia erecta*. J. Nat. Prod..

[4] Sun L.-L., Li W.-S., Li J., Zhang H.-Y., Yao L.-G., Luo H., Guo Y.-W., Li X.-W. (2021). Uncommon Diterpenoids from the South China Sea Soft Coral *Sinularia humilis* and Their Stereochemistry. J. Org. Chem..

[5] Yang M., Cui W.-X., Li H., Tang W., Li S.-W., Yao L.-G., Mudianta I.W., Guo Y.-W. (2020). Sinulasterols A-C, three new bioactive oxygenated steroids from the South China Sea soft coral *Sinularia depressa*. Steroids.

[6] Tammam M.A., Rarova L., Kvasnicova M., Emam A.M., Gonzalez G., Mahdy A., Strand M., Ioannou E., Roussis V. (2020). Bioactive Steroids from the Red Sea Soft Coral *Sinularia polydactyla*. Mar. Drugs.

[7] Liu J., Li H., Wu M.-J., Tang W., Wang J.-R., Gu Y.-C., Wang H., Li X.-W., Guo Y.-W. (2020). Sinueretone A, a diterpenoid with unprecedented tricyclo[12.1.0.0(5,9)]pentadecane carbon scaffold from the South China Sea soft coral *Sinularia erecta*. J. Org. Chem..

[8] Cai Y.-S., Cui W.-X., Tang W., Guo Y.-W. (2020). Uncommon terpenoids with anti-inflammatory activity from the Hainan soft coral *Sinularia tumulosa*. Bioorg. Chem..

[9] Jiang C.-S., Ru T., Huan X.-J., Miao Z.-H., Guo Y.-W. (2019). New cytotoxic ergostane-type sterols from the Chinese soft coral *Sinularia* sp.. Steroids.

[10] Qin G.-F., Tang X.-L., Sun Y.-T., Luo X.-C., Zhang J., Ofwegen L.V., Sung P.-J., Li P.-L., Li G.-Q. (2018). Terpenoids from the Soft Coral *Sinularia* sp. Collected in Yongxing Island. Mar. Drugs.

[11] Li S.-W., Chen W.-T., Yao L.-G., Guo Y.-W. (2018). Two new cytotoxic steroids from the Chinese soft coral *Sinularia* sp.. Steroids.

[12] Huang C.-Y., Su J.-H., Liaw C.-C., Sung P.-J., Chiang P.-L., Hwang T.-L., Dai C.-F., Sheu J.-H. (2017). Bioactive Steroids with Methyl Ester Group in the Side Chain from a Reef Soft Coral *Sinularia brassica* Cultured in a Tank. Mar. Drugs.

[13] Thao N.P., Nam N.H., Cuong N.X., Quang T.H., Tung P.T., Dat L.D., Chae D., Kim S., Koh Y.-S., Van Kiem P. (2013). Anti-inflammatory norditerpenoids from the soft coral *Sinularia maxima*. Bioorg. Med. Chem. Lett..

[14] Lai D., Geng Z., Deng Z., Van Ofwegen L., Proksch P., Lin W. (2013). Cembranoids from the Soft Coral *Sinularia* rigida with Antifouling Activities. J. Agric. Food Chem..

[15] Liang L.-F., Li Y.-F., Liu H.-L., Guo Y.-W. (2013). Research advance on the chemistry and bioactivity of secondary metabolites from the soft corals of the genus *Sinularia*. Guoji Yaoxue Yanjiu Zazhi.

[16] Sheu J.-H., Peng B.-R., Fang L.-S., Hwang T.-L., Su J.-H., Wu Y.-C., Sung P.-J. (2019). Hydroperoxyditerpenoids from Octocorals. Isr. J. Chem..

[17] Lakshmi V., Kumar R. (2009). Metabolites from *Sinularia* species. Nat. Prod. Res..

[18] Rodrigues I.G., Miguel M.G., Mnif W. (2019). A brief review on new naturally occurring cembranoid diterpene derivatives from the soft corals of the Genera *Sarcophyton*, *Sinularia*, and *Lobophytum* since 2016. Molecules.

[19] Mohammadi P.P., Yegdaneh A., Aghaei M., Ali Z., Khan I.A., Ghanadian M. (2021). Novel 16,17-epoxy-23-methylergostane derivative from *Sinularia variabilis*, a soft coral from the Persian Gulf, with apoptotic activities against breast cancer cell lines. Nat. Prod. Res..

[20] Lu Y.-H., Lin J.-J., Wu Y.-J., Su J.-H., El-Shazly M. (2021). Quinone Derivatives from the Soft Coral *Sinularia* scabra. Chem. Nat. Compd..

[21] Ye F., Chen Z.-H., Gu Y.-C., Guo Y.-W., Li X.-W. (2020). New lobane-type diterpenoids from the Xisha soft coral *Sinularia polydactyla*. Chin. J. Nat. Med..

[22] Yang M., Liang L.-F., Li H., Tang W., Guo Y.-W. (2020). A new 5α,8α-epidioxysterol with immunosuppressive activity from the South China Sea soft coral *Sinularia* sp.. Nat. Prod. Res..

[23] Li J., Huan X.-J., Wu M.-J., Chen Z.-H., Chen B., Miao Z.-H., Guo Y.-W., Li X.-W. (2020). Chemical constituents from the South China sea soft coral *Sinularia humilis*. Nat. Prod. Res..

[24] Cui W.-X., Yang M., Li G., Li H., Tang W., Li S.-W., Yao L.-G., Wang C.-H., Liang L.-F., Guo Y.-W. (2020). Polycyclic furanobutenolide-derived norditerpenoids from the South China Sea soft corals *Sinularia scabra* and *Sinularia polydactyla* with immunosuppressive activity. Bioorg. Chem..

[25] Chang Y.-C., Lai K.-H., Kumar S., Chen P.-J., Wu Y.-H., Lai C.-L., Hsieh H.-L., Sung P.-J., Hwang T.-L. (2020). NMR H^1^-based isolation of anti-inflammatory 9,11-secosteroids from the octocoral *Sinularia leptoclados*. Mar. Drugs.

[26] Yang M., Li G., Li H., Zhang Q., Wu Q.-H., Chen K.-X., Guo Y.-W., Li X.-W., Tang W. (2019). Highly diverse cembranoids from the South China Sea soft coral *Sinularia scabra* as a new class of potential immunosuppressive agents. Bioorg. Med. Chem..

[27] Wu Q., Li X.-W., Li H., Yao L.-G., Tang W., Miao Z.-H., Wang H., Guo Y.-W. (2019). Bioactive polyoxygenated cembranoids from a novel Hainan chemotype of the soft coral *Sinularia flexibilis*. Bioorg. Med. Chem. Lett..

[28] Wang Z., Li P.-L., Luo X.-C., Wang Q., Ofwegen L.V., Tang X.L., Li G.-Q. (2019). Terpenoids from the South China Sea soft coral *Sinularia multiflora*. Nat. Prod. Res..

[29] Phan C.-S., Yee C.-S., Vairappan C.S., Ishii T., Kamada T. (2019). Sinulaflexiolide P, A Cembrane-Type Diterpenoid from Bornean Soft Coral *Sinularia flexibilis*. Chem. Nat. Compd..

[30] Lu S.-Q., Li X.-W., Li S.-W., Cui Z., Guo Y.-W., Han G.-Y. (2019). Sinuhirtins A and B, two uncommon norhumulene-type terpenoids from the South China Sea soft coral *Sinularia hirta*. Tetrahedron Lett..

[31] Lai W., Zou G., Liao X.-J., Zhang H., Zhao B.-X., Xu S.-H., Qin S.-Y., Chen G.-D. (2019). Sinulaspirolactam A, a novel aza-spirocyclic valerenane sesquiterpenoid from soft coral *Sinularia* sp.. J. Asian Nat. Prod. Res..

[32] Jiang C.-S., Ru T., Yao L.-G., Miao Z.-H., Guo Y.-W. (2019). Four new cembranoids from the Chinese soft coral *Sinularia* sp. and their anti-Aβ aggregation activities. Fitoterapia.

[33] Huong N.T., Ngoc N.T., Hanh T.T.H., Quang T.H., Cuong N.X., Nam N.H., Van Minh C. (2019). Chemical constituents from the soft coral *Sinularia digitata*. Vietnam. J. Chem..

[34] Ye F., Zhu Z.-D., Gu Y.C., Li J., Zhu W.-L., Guo Y.-W. (2018). Further New Diterpenoids as PTP1B Inhibitors from the Xisha Soft Coral *Sinularia polydactyla*. Mar. Drugs.

[35] Wu C.-H., Chao C.-H., Huang T.-Z., Huang C.-Y., Hwang T.-L., Dai C.-F., Sheu J.-H. (2018). Cembranoid-Related Metabolites and Biological Activities from the Soft Coral *Sinularia flexibilis*. Mar. Drugs.

[36] Kamada T., Phan C.-S., Zanil I.I., Vairappan C.S., Kang M.-C., Jeon Y.-J. (2018). Bioactive cembranoids from the soft coral Genus *Sinularia* sp. in Borneo. Mar. Drugs.

[37] Kamada T., Phan C.-S., Hamada T., Hatai K., Vairappan C.S. (2018). Cytotoxic and Antifungal Terpenoids from Bornean Soft Coral, *Sinularia flexibilis*. Nat. Prod. Commun..

[38] Huong N.T., Ngoc N.T., Thanh N.V., Dang N.H., Cuong N.X., Nam N.H., Kiem P.V., Minh C.V., Cuong N.X., Thung D.C. (2018). Eudesmane and aromadendrane sesquiterpenoids from the Vietnamese soft coral *Sinularia erecta*. Nat. Prod. Res..

[39] Hsu F.-Y., Duh C.-Y., Wang S.-K. (2018). Xeniaphyllane-Derived Terpenoids from Soft Coral *Sinularia nanolobata*. Mar. Drugs.

[40] Chu M.-J., Tang X.-L., Han X., Li T., Luo X.-C., Jiang M.-M., Van Ofwegen L., Luo L.-Z., Zhang G., Li P.-L. (2018). Metabolites from the Paracel Islands Soft Coral *Sinularia* cf. molesta. Mar. Drugs.

[41] Ye F., Zhu Z.-D., Chen J.-S., Li J., Gu Y.-C., Zhu W.-L., Li X.-W., Guo Y.-W. (2017). Xishacorenes A–C, Diterpenes with Bicyclo[3.3.1]nonane Nucleus from the Xisha Soft Coral *Sinularia polydactyla*. Org. Lett..

[42] Wang J., Su P., Gu Q., Li W.D., Guo J.L., Qiao W., Feng D.Q., Tang S.A. (2017). Antifouling activity against bryozoan and barnacle by cembrane diterpenes from the soft coral *Sinularia flexibilis*. Int. Biodeterior. Biodegrad..

[43] Ngoc N.T., Hanh T.T.H., Nguyen V.T., Thao D.T., Cuong N.X., Nguyen H.N., Thung D.C., Kiem P.V., Minh C.V. (2017). Cytotoxic steroid derivatives from the Vietnamese soft coral *Sinularia brassica*. J. Asian Nat. Prod. Res..

[44] Ngoc N.T., Huong P.T.M., Thanh N.V., Cuong N.X., Nam N.H., Thung D.C., Kiem P.V., Minh C.V. (2017). Sesquiterpene constituents from the soft coral *Sinularia nanolobata*. Nat. Prod. Res..

[45] Ngoc N.T., Huong P.T.M., Thanh N.V., Chi N.T.P., Dang N.H., Cuong N.X., Nam N.H., Thung D.C., Kiem P.V., Minh C.V. (2017). Cytotoxic Steroids from the Vietnamese Soft Coral *Sinularia conferta*. Chem. Pharm. Bull..

[46] Ngoc N.T., Hanh T.T.H., Thanh N.V., Thao D.T., Cuong N.X., Nam N.H., Thung D.C., Kiem P.V., Minh C.V. (2017). Cytotoxic steroids from the Vietnamese soft coral *Sinularia leptoclados*. Chem. Pharm. Bull..

[47] Mohammed R., Radwan M.M., Ma G., Mohamed T.A., Seliem M.A., Thabet M., ElSohly M.A. (2017). Bioactive sterols and sesquiterpenes from the Red Sea soft coral *Sinularia terspilli*. Med. Chem. Res..

[48] Huang C.-Y., Ahmed A.F., Su J.-H., Sung P.-J., Hwang T.-L., Chiang P.-L., Dai C.-F., Liaw C.-C., Sheu J.-H. (2017). Bioactive new withanolides from the cultured soft coral *Sinularia brassica*. Bioorg. Med. Chem. Lett..

[49] Yuan W., Cheng S., Fu W., Zhao M., Li X., Cai Y., Dong J., Huang K., Gustafson K.R., Yan P. (2016). Structurally diverse metabolites from the soft coral *Sinularia verruca* collected in the South China Sea. J. Nat. Prod..

[50] Sun H., Liu F., Feng M.-R., Peng Q., Liao X.-J., Liu T.-T., Xu S.-H., Zhang J. (2016). Isolation of a new cytotoxic polyhydroxysterol from the South China Sea soft coral *Sinularia* sp.. Nat. Prod. Res..

[51] Roy P.K., Ashimine R., Ueda K., Miyazato H., Taira J. (2016). Endoperoxy and hydroperoxy cadinane-type sesquiterpenoids from an Okinawan soft coral, *Sinularia* sp.. Arch. Pharmacal Res..

[52] Rahelivao M.P., Gruner M., Lübken T., Islamov D., Kataeva O., Andriamanantoanina H., Bauer I., Knölker H.-J. (2016). Chemical constituents of the soft corals *Sinularia vanderlandi* and *Sinularia gravis* from the coast of Madagascar. Org. Biomol. Chem..

[53] Phan C.-S., Ng S.Y., Kamada T., Vairappan C.S. (2016). Two New Lobane Diterpenes from a Bornean Soft Coral *Sinularia* sp.. Nat. Prod. Commun..

[54] Nguyen V.T., Ngoc N.T., Anh H.L.T., Thung D.C., Thao D.T., Nguyen X.C., Nguyen H.N., Kiem P.V., Minh C.V. (2016). Steroid constituents from the soft coral *Sinularia microspiculata*. J. Asian Nat. Prod. Res..

[55] Ngoc N.T., Huong P.T.M., Thanh N.V., Cuong N.X., Nam N.H., Thung D.C., Kiem P.V., Minh C.V. (2016). Steroid Constituents from the Soft Coral *Sinularia nanolobata*. Chem. Pharm. Bull..

[56] Lin Y.-S., Su J.-H., Lo C.-L., Huang C.-Y., Sheu J.-H. (2016). Isobicyclogermacrene-type Sesquiterpenoids from the Soft Coral *Sinularia lochmodes*. Nat. Prod. Commun..

[57] Hegazy M.-E.F., Mohamed T.A., ElShamy A.I., Al-Hammady M.A., Ohta S., Paré P.W. (2016). Casbane Diterpenes from Red Sea Coral *Sinularia polydactyla*. Molecules.

[58] Chitturi B.R., Tatipamula V.B., Dokuburra C.B., Mangamuri U.K., Tuniki V.R., Kalivendi S.V., Bunce R.A., Yenamandra V. (2016). Pambanolides A–C from the South Indian soft coral *Sinularia inelegans*. Tetrahedron.

[59] Chao C.-H., Wu C.-Y., Huang C.-Y., Wang H.-C., Dai C.-F., Wu Y.-C., Sheu J.-H. (2016). Cubitanoids and Cembranoids from the Soft Coral *Sinularia nanolobata*. Mar. Drugs.

[60] Zhang N.-X., Tang X.-L., van Ofwegen L., Xue L., Song W.-J., Li P.-L., Li G.-Q. (2015). Cyclopentenone derivatives and polyhydroxylated steroids from the soft coral *Sinularia acuta*. Chem. Biodivers..

[61] Yin C.-T., Wen Z.-H., Lan Y.-H., Chang Y.-C., Wu Y.-C., Sung P.-J. (2015). New Anti-inflammatory Norcembranoids from the Soft Coral *Sinularia numerosa*. Chem. Pharm. Bull..

[62] Yang B., Huang J., Lin X., Liao S., Zhou X., Liu J., Wang J., Wang L., Liu Y. (2015). New Casbane Diterpenoids from the Hainan Soft Coral *Sinularia* Species. Helv. Chim. Acta.

[63] Tsai T.-C., Chen H.-Y., Sheu J.-H., Chiang M.Y., Wen Z.-H., Dai C.-F., Su J.-H. (2015). Structural Elucidation and Structure–Anti-inflammatory Activity Relationships of Cembranoids from Cultured Soft Corals *Sinularia* sandensis and *Sinularia flexibilis*. J. Agric. Food Chem..

[64] Lin W.-J., Wu T.-Y., Su T.-R., Wen Z.-H., Chen J.-J., Fang L.-S., Wu Y.-C., Sung P.-J. (2015). Terpenoids from the octocoral *Sinularia gaweli*. Int. J. Mol. Sci..

[65] Chen W.-T., Li J., Wang J.-R., Li X.-W., Guo Y.-W. (2015). Structural diversity of terpenoids in the soft coral *Sinularia flexibilis*, evidenced by a collection from the South China Sea. RSC Adv..

[66] Chen W.-F., Yin C.-T., Cheng C.-H., Lu M.-C., Fang L.-S., Wang W.-H., Wen Z.-H., Chen J.-J., Wu Y.-C., Sung P.-J. (2015). Norcembranoidal Diterpenes from the Cultured-Type Octocoral *Sinularia numerosa*. Int. J. Mol. Sci..

[67] Chao C.-H., Huang T.-Z., Wu C.-Y., Chen B.-W., Huang C.-Y., Hwang T.-L., Dai C.-F., Sheu J.-H. (2015). Steroidal and α-tocopherylhydroquinone glycosides from two soft corals Cladiella hirsuta and *Sinularia nanolobata*. RSC Adv..

[68] Yang B., Lin X., Liu J., Liao S., Wang J., Zhou X., Liu Y., Wei X., Huang J., Wang L. (2014). Sinulolides A-H, new cyclopentenone and butenolide derivatives from soft coral *Sinularia* sp.. Mar. Drugs.

[69] Wang L.-H., Chen K.-H., Dai C.-F., Hwang T.-L., Wang W.-H., Wen Z.-H., Wu Y.-C., Sung P.-J. (2014). New Cembranoids from the Soft Coral *Sinularia arborea*. Nat. Prod. Commun..

[70] Rajaram S., Ramesh D., Ramulu U., Anjum M., Kumar P., Murthy U.S.N., Hussain M.A., Sastry G.N., Venkateswarlu Y. (2014). Chemical examination of the soft coral *Sinularia kavarattiensis* and evaluation of anti-microbial activity. Indian J. Chem. Sect. B Org. Chem. Incl. Med. Chem..

[71] Lillsunde K.-E., Festa C., Adel H., De Marino S., Lombardi V., Tilvi S., Nawrot D.A., Zampella A., D’Souza L., D’Auria M.V. (2014). Bioactive Cembrane Derivatives from the Indian Ocean Soft Coral, *Sinularia kavarattiensis*. Mar. Drugs.

[72] Lei L.-F., Chen M.-F., Wang T., He X.-X., Liu B.-X., Deng Y., Chen X.-J., Li Y.-T., Guan S.-Y., Yao J.-H. (2014). Novel cytotoxic nine-membered macrocyclic polysulfur cembranoid lactones from the soft coral *Sinularia* sp.. Tetrahedron.

[73] Jiang C.-S., Li Y., Han G.-Y., Guo Y.-W. (2014). Further casbane-type diterpenes from the soft coral *Sinularia depressa*. Zhongguo Tianran Yaowu.

[74] Chen W.-T., Liu H.-L., Yao L.-G., Guo Y.-W. (2014). 9,11-Secosteroids and polyhydroxylated steroids from two South China Sea soft corals Sarcophyton trocheliophorum and *Sinularia flexibilis*. Steroids.

[75] Chen K.-H., Dai C.-F., Hwang T.-L., Chen C.-Y., Li J.-J., Chen J.-J., Wu Y.-C., Sheu J.-H., Wang W.-H., Sung P.-J. (2014). Discovery of novel diterpenoids from *Sinularia arborea*. Mar. Drugs.

[76] Chen D., Cheng W., Liu D., Van Ofwegen L., Proksch P., Lin W. (2014). Capillosananes S–Z, new sesquiterpenoids from the soft coral *Sinularia capillosa*. Tetrahedron Lett..

[77] Yin J., Zhao M., Ma M., Xu Y., Xiang Z., Cai Y., Dong J., Lei X., Huang K., Yan P. (2013). New Casbane Diterpenoids from a South China Sea Soft Coral, *Sinularia* sp.. Mar. Drugs.

[78] Yang B., Liao S., Lin X., Wang J., Liu J., Zhou X., Yang X., Liu Y. (2013). New Sinularianin sesquiterpenes from soft coral *Sinularia* sp.. Mar. Drugs.

[79] Tsai T.-C., Wu Y.-J., Su J.-H., Lin W.-T., Lin Y.-S. (2013). A new spatane diterpenoid from the cultured soft coral *Sinularia leptoclados*. Mar. Drugs.

[80] Shaaban M., Shaaban K.A., Ghani M.A. (2013). Hurgadacin: A new steroid from *Sinularia polydactyla*. Steroids.

[81] Rajaram S., Ramulu U., Ramesh D., Srikanth D., Bhattacharya P., Prabhakar P., Kalivendi S.V., Babu K.S., Venkateswarlu Y., Navath S. (2013). Anti-cancer evaluation of carboxamides of furano-sesquiterpene carboxylic acids from the soft coral *Sinularia kavarattiensis*. Bioorg. Med. Chem. Lett..

[82] Nguyen P.T., Nguyen H.N., Nguyen X.C., Tai B.H., Quang T.H., Nguyen T.T.N., Luyen B.T.T., Yang S.Y., Choi C.H., Kim S. (2013). Steroidal constituents from the soft coral *Sinularia dissecta* and their inhibitory effects on lipopolysaccharide-stimulated production of pro-inflammatory cytokines in bone marrow-derived dendritic cells. Bull. Korean Chem. Soc..

[83] Liang L.-F., Wang X.-J., Zhang H.-Y., Liu H.-L., Li J., Lan L.-F., Zhang W., Guo Y.-W. (2013). Bioactive polyhydroxylated steroids from the Hainan soft coral *Sinularia depressa* Tixier-Durivault. Bioorg. Med. Chem. Lett..

[84] Huang C.-Y., Liaw C.-C., Chen B.-W., Chen P.-C., Su J.-H., Sung P.-J., Dai C.-F., Chiang M.Y., Sheu J.-H. (2013). Withanolide-Based Steroids from the Cultured Soft Coral *Sinularia brassica*. J. Nat. Prod..

[85] Hu L.-C., Yen W.-H., Su J.-H., Chiang M.Y.-N., Wen Z.-H., Chen W.-F., Lu T.-J., Chang Y.-W., Chen Y.-H., Wang W.-H. (2013). Cembrane Derivatives from the Soft Corals, *Sinularia gaweli* and *Sinularia flexibilis*. Mar. Drugs.

[86] Ahmed S., Ibrahim A., Arafa A.S. (2013). Anti-H5N1 virus metabolites from the Red Sea soft coral, *Sinularia candidula*. Tetrahedron Lett..

[87] Aboutabl E.-S.A., Azzam S.M., Michel C.G., Selim N.M., Hegazy M.F., Ali A.-H.A.M., Hussein A.A. (2013). Bioactive terpenoids from the Red Sea soft coral *Sinularia polydactyla*. Nat. Prod. Res..

[88] Wu M.-J., Wang H., Jiang C.-S., Guo Y.-W. (2020). New cembrane-type diterpenoids from the South China Sea soft coral *Sinularia crassa* and their α-glucosidase inhibitory activity. Bioorg. Chem..

[89] Khushi S., Salim A.A., Elbanna A.H., Nahar L., Bernhardt P.V., Capon R.J. (2020). Dysidealactams and dysidealactones: Sesquiterpene glycinyl-lactams, imides, and lactones from a *Dysidea* sp. marine sponge collected in southern Australia. J. Nat. Prod..

[90] Imperatore C., Gimmelli R., Persico M., Casertano M., Guidi A., Saccoccia F., Ruberti G., Luciano P., Aiello A., Parapini S. (2020). Investigating the Antiparasitic Potential of the Marine Sesquiterpene Avarone, Its Reduced Form Avarol, and the Novel Semisynthetic Thiazinoquinone Analogue Thiazoavarone. Mar. Drugs.

[91] Rosa S.D., Iodice C., Khalaghdoust M., Oryan S., Rustaiyan A. (1999). Spatane diterpenoids from the brown alga *Stoechospermum marginatum* (Dictyotaceae). Phytochemistry.

[92] Lin Y.-F., Kuo C.-Y., Wen Z.-H., Lin Y.-Y., Wang W.-H., Su J.-H., Sheu J.-H., Sung P.-J. (2013). Flexibilisquinone, a new anti-inflammatory quinone from the cultured soft coral *Sinularia flexibilis*. Molecules.

[93] Tursch B., Braekman J.C., Daloze D., Herin M., Karlsson R., Losman D. (1975). Chemical studies of marine invertebrates. XI. Sinulariolide, a new cembranolide diterpene from the soft coral *Sinularia flexibilis*. Tetrahedron.

[94] Casertano M., Menna M., Imperatore C. (2020). The Ascidian-Derived Metabolites with Antimicrobial Properties. Antibiotics.

[95] Li J., Tao H., Lei X.-X., Zhang H., Zhou X., Liu Y., Li Y., Yang B. (2021). Arthriniumsteroids A–D, four new steroids from the soft coral-derived fungus *Simplicillium lanosoniveum* SCSIO41212. Steroids.

[96] Xu W.-F., Chao R., Hai Y., Guo Y.-Y., Wei M.-Y., Wang C.-Y., Shao C.-L. (2021). 17-Hydroxybrevianamide N and Its N1-Methyl Derivative, Quinazolinones from a Soft-Coral-Derived *Aspergillus* sp. Fungus: 13S Enantiomers as the True Natural Products. J. Nat. Prod..

[97] Pham T.M., Wiese J., Wenzel-Storjohann A., Imhoff J.F. (2016). Diversity and antimicrobial potential of bacterial isolates associated with the soft coral *Alcyonium digitatum* from the Baltic Sea. Anton. Leeuw..

[98] Yang S., Sun W., Tang C., Jin L., Zhang F., Li Z. (2013). Phylogenetic diversity of actinobacteria associated with soft coral *Alcyonium gracllimum* and stony coral *Tubastraea coccinea* in the East China Sea. Microb. Ecol..

